# Toxic traditional Chinese medicines as hormetic immunomodulators in cancer: from toxicity-efficacy homology to translational opportunities

**DOI:** 10.3389/fimmu.2026.1965726

**Published:** 2026-10-19

**Authors:** Qing Tong, Zhiqiang Cai

**Affiliations:** 1 Department of Oncology, The First Affiliated Hospital of Yangtze University, Jingzhou, China; 2 Department of Pharmacology, Health Science Center, Yangtze University, Jingzhou, China

**Keywords:** clinical translation, combination therapy, hormesis, toxic traditional Chinese medicines, toxicity-efficacy homology, tumor immune microenvironment

## Abstract

The immunosuppressive tumor microenvironment (TIME) remains a major barrier to cancer immunotherapy, creating an urgent need for agents that can simultaneously remodel the TIME and potentiate immune checkpoint blockade. In this review, we propose the concept of “toxicity-efficacy homology”, which holds that the reactive chemical moieties of toxic traditional Chinese medicines (TCMs) serve as the shared structural basis for both their immunomodulatory activity and organ toxicity. Within a hormetic framework, these agents activate anti-tumor immunity within a low-dose “immunoactivation window”, whereas higher doses elicit non-specific toxicity. This review examines the molecular mechanisms, preclinical evidence, and clinical data for representative toxic TCMs, and highlights three interrelated translational bottlenecks: a narrow therapeutic window, low bioavailability, and quality-control challenges. To address these obstacles, a solution framework is outlined that integrates structural modification and prodrug design, intelligent nano-delivery, traditional compatibility-based detoxification, and a precision combination strategy guided by tumor immune phenotyping. Collectively, this review provides a conceptual basis for repositioning toxic TCMs from empirical “combating poison with poison” into mechanism-driven, evidence-based “attenuated toxicity and enhanced efficacy” immunotherapeutic agents.

## 1 Introduction

ICIs have transformed cancer therapy, but objective response rates in most solid tumors remain only 20-40%, and resistance is associated with defective antigen presentation, insufficient effector T-cell infiltration, compensatory checkpoint upregulation, metabolic dysfunction, and physical exclusion of T cells by dense or hypoxic stroma (1–7); ICIs can also cause immune-related adverse events (8), underscoring the urgent need for agents that remodel the TIME and potentiate ICIs with acceptable safety (9). Toxic TCMs, guided by the principle of “combating poison with poison”, have long been applied to “abdominal masses”, toxin accumulation, and pain (10–12), and modern research indicates that their antitumor effects arise from both direct cytotoxicity and multi-target immunomodulation. Representative components such as ATO, cantharidin, cinobufagin, and icaritin induce ICD, promote CD8^+^ T-cell infiltration, or regulate PD-L1, suggesting that these agents may act as dose-dependent immunomodulators rather than merely cytotoxic agents (13–18). Toxic TCMs are defined operationally as those meeting at least two of six predefined criteria (pharmacopoeial toxicity, toxic moieties, narrow therapeutic window, need for processing, clinical poisoning reports, or preclinical organ toxicity) (19–21).

A recurring feature of many toxic TCMs is that the same chemical moiety or structural domain can contribute to both immunomodulatory activity and organ toxicity. This phenomenon is referred to here as “toxicity-efficacy homology”. It denotes a structurally defined overlap that is testable by structure-activity relationship studies, chemical modification, prodrug design, or detoxification processing. Several core components illustrate this concept. The anhydride ring of cantharidin inhibits PP2A and induces ICD, while covalent binding to biological amines contributes to nephrotoxicity (22, 23). The C8-C14 diester bonds of aconitine mediate sodium channel activation and cardiotonic effects but also cause cardiotoxicity and neurotoxicity (24, 25). The triepoxide structure of triptolide inhibits RNA polymerase II via covalent binding to the XPB subunit and induces apoptosis, but also contributes to hepatotoxicity and reproductive toxicity (11, 26). The enolide ring of bufadienolides inhibits Na^+^/K^+^-ATPase and induces tumor cell death, while also causing cardiotoxicity (27). The N→O coordination bond of oxymatrine acts as a mild ROS inducer at low doses and participates in epigenetic regulation, including DNA demethylation-mediated PD-L1 downregulation, whereas high doses induce oxidative damage (28). This overlap is chemically separable: norcantharidin retains antitumor activity with reduced nephrotoxicity (29), and in Aconitum carmichaelii (Fuzi), toxic diester diterpenoid alkaloids coexist with immunoprotective neutral polysaccharides (30). Low-toxicity Epimedium flavonoids and centipede peptides are included as boundary cases because they lack strong electrophilic groups and achieve efficacy/toxicity balance mainly through dose-dependent signaling modulation (31, 32).

Hormesis describes a biphasic dose-response in which low doses stimulate and high doses inhibit or cause toxicity (33–35). In toxic TCM research, the same agent may enhance immune activation at low doses while producing organ toxicity at higher doses. The term “immunoactivation window” is used here to denote a dose range in which immune activation is increased and toxicity remains tolerable. This window lies within the therapeutic window and provides a practical framework for dose selection. Arsenic, cantharidin, arenobufagin, and cinobufagin each show dose-dependent immunomodulation, with immune activation at lower doses and organ toxicity at higher doses.At low doses, arsenic induces ER stress, ROS burst, and immune activation, whereas higher doses cause apoptosis and organ damage (14, 15). Cantharidin promotes ICD and T-cell infiltration at low doses but causes renal tubular damage at higher doses (16, 23). Arenobufagin modulates the HSP90β-STAT3-PD-L1 axis at low concentrations but is directly cytotoxic at high concentrations (36). Cinobufagin induces immunogenic PANoptosis and T-cell infiltration at lower doses, inhibits tumor growth at intermediate doses, and shows mild histopathological changes at higher doses (17, 37, 38). These observations support a dose range in which immune activation predominates over toxicity, although systematic dose-response mapping is still required.

In this review, we examine how chemical features link immunomodulation and toxicity, whether an immunoactivation window can be defined for representative components, how structural modification, delivery systems, compatibility, and processing can reduce toxicity, and how toxic TCM components may be combined with ICIs, chemotherapy, radiotherapy, and potentially CAR-T therapy. The integrated framework that links toxicity-efficacy homology, the operational immunoactivation window, TIME-remodeling mechanisms, and translational strategies, is shown in **Figure 1**.

**Figure 1:**
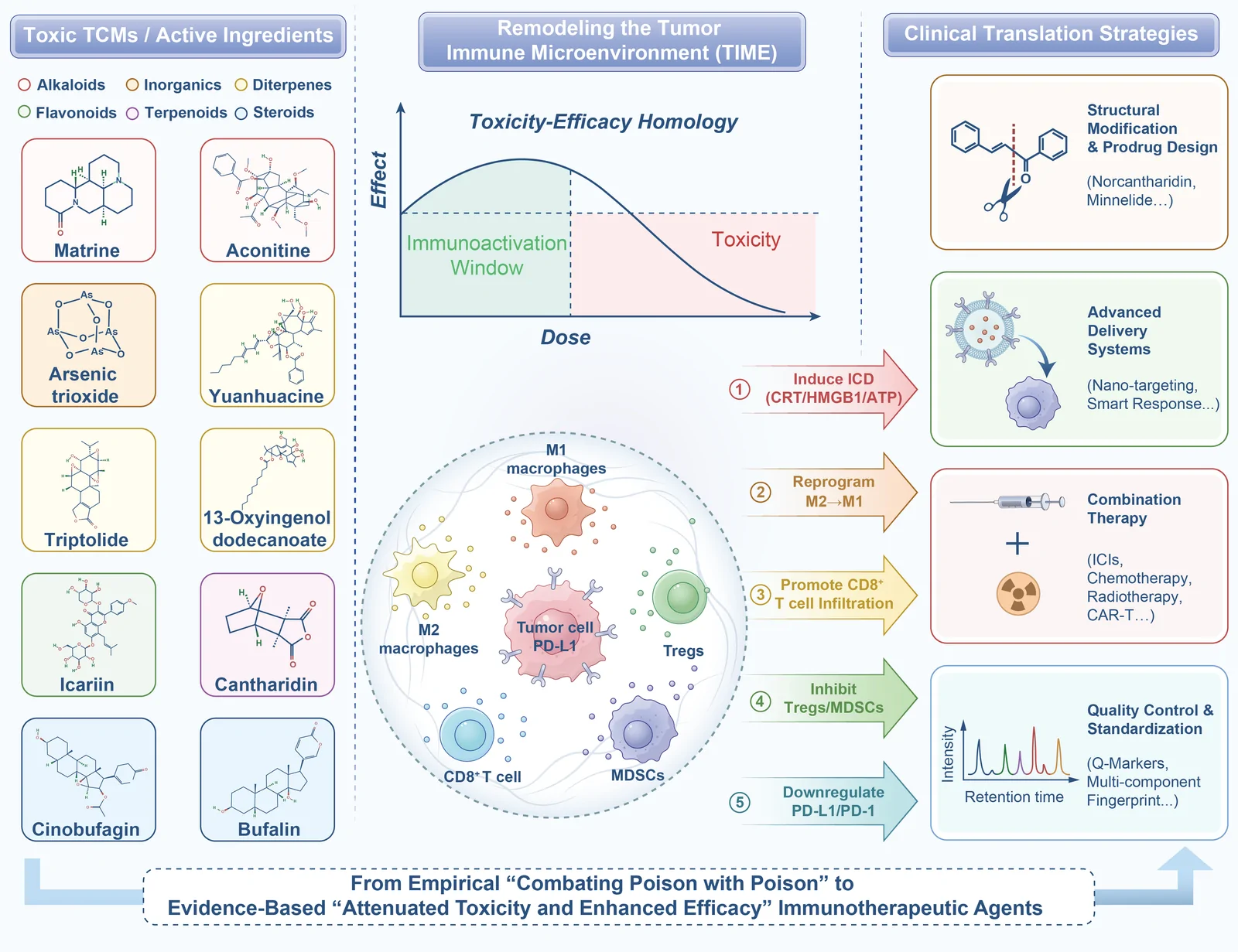
Integrated framework of toxicity-efficacy homology, the operational immunoactivation window, TIME-remodeling mechanisms, and clinical translation strategies for toxic TCMs. The figure illustrates: (i) representative active components and their shared toxicity-efficacy homology chemical groups, including anhydride, epoxide, enolide, diester, and α, β-unsaturated carbonyl moieties; (ii) the conceptual dose-immunoactivation-toxicity windows, with the immunoactivation window defined as an operational dose range within the therapeutic window; (iii) the core mechanisms of TIME remodeling, including ICD/RCD induction, TAM reprogramming, CD8^+^ T-cell infiltration, Treg/MDSC inhibition, and PD-L1 downregulation; and (iv) four clinical translation strategies, including structural modification and prodrug design, advanced delivery systems, combination therapy, and quality control and standardization.

## 2 Core toxic TCMs: chemical basis and toxicity-efficacy homology

### 2.1 Representative toxic TCMs

The core toxic TCMs discussed in this review are Mylabris (cantharidin), Bufonis Venenum (bufadienolides), Arsenicum Trioxydum (ATO), Tripterygium wilfordii (triptolide, celastrol, triptonide), Aconitum carmichaelii (aconitine, mesaconitine, benzoylaconine), Euphorbia kansui (ingenane and jatrophane diterpenoids), Sophora flavescens (matrine, oxymatrine, kurarinone), and Genkwa Flos (yuanhuacine, yuanhuadine). Epimedium and Scolopendra are discussed only as boundary cases. As shown in **Table 1**, these agents contain characteristic toxicity-efficacy homology groups, including anhydride, epoxide, enolide, diester, and α, β-unsaturated carbonyl moieties.

**Table 1:** Core toxic TCMs and their toxicity-efficacy homology groups.

TCM	Representative active components	Chemical class	Toxicity-efficacy homology group	Immunological activity	Major organ toxicity	References
*Mylabris* (Spanish fly)	Cantharidin, norcantharidin	Terpenoid anhydride	Anhydride ring	PP2A inhibition, ICD induction	Nephrotoxicity, hepatotoxicity, cardiotoxicity	(22, 23, 29, 39, 40, 59, 60, 64)
*Bufonis Venenum* (toad venom)	Cinobufagin, bufalin, arenobufagin	Bufadienolide (steroid)	Enolide lactone ring	Na^+^/K^+^-ATPase inhibition, ICD, TAM reprogramming	Cardiotoxicity, neurotoxicity	(27, 36–38, 41, 75, 76, 86)
*Arsenicum Trioxydum* (arsenic trioxide, ATO)	As_2_O_3_ (As^III^)	Inorganic compound	Trivalent arsenic (electrophilic)	cGAS-STING activation, ICD, M1 polarization	Hepatotoxicity, nephrotoxicity, neurotoxicity	(13–15, 99, 105, 110)
*Tripterygium wilfordii* (Thunder god vine)	Triptolide, celastrol, triptonide	Diterpenoid epoxide / triterpenoid	Triepoxide, α,β-unsaturated ketone	RNA pol II inhibition, Treg inhibition, PD-L1 downregulation	Hepatotoxicity, reproductive toxicity, cardiotoxicity	(11, 26, 78, 95)
*Aconitum carmichaelii* (Fuzi)	Aconitine, mesaconitine, benzoylaconine	Diterpenoid alkaloid	C8-C14 diester bond (or monoester)	Sodium channel activation, immunomodulation	Cardiotoxicity, neurotoxicity	(24, 25, 42, 51, 52, 61, 62)
*Euphorbia kansui* (Kansui)	13-Oxyingenol dodecanoate, ingenane-type diterpenoids	Diterpenoid (ingenane/jatrophane)	Epoxide, unsaturated ester	TMBIM6 targeting, mitophagy, ferroptosis, MDR reversal	Gastrointestinal toxicity, hepatotoxicity	(44, 142, 179)
*Sophora flavescens* (Kushen)	Matrine, oxymatrine, kurarinone	Quinolizidine alkaloid / flavonoid	N→O coordination bond (oxymatrine)	PI3K/AKT/STAT3 inhibition, DNA demethylation, PD-L1 downregulation	Low toxicity; cardiac and neurological toxicity at high doses	(28, 48–50, 91, 122, 171, 178)
*Genkwa Flos* (Yuanhua)	Yuanhuacine, yuanhuadine	Diterpenoid (daphnane)	α,β-Unsaturated carbonyl	PKC activation, cytokine modulation	Gastrointestinal toxicity	(43, 138)
*Scolopendra* (centipede) — boundary case	Scolopentide, polysaccharide-protein complex (SPPC)	Peptide, polysaccharide	No strong electrophilic group	DR4/DR5 binding, NK/CTL activation	Neurotoxicity, allergy at high doses	(32)
*Epimedium* (Yinyanghuo) — boundary case	Icariin, icaritin, icariside I, epimedokoreanin B	8-Prenylflavonoid	No strong electrophilic group	IKK-α/NF-κB inhibition, pyroptosis, ferroptosis, kynurenine-AhR blockade	Low toxicity	(18, 31, 56, 67, 83, 87, 89)

### 2.2 Electrophilic groups: anhydrides, epoxides, and enolides

Electrophilic groups in several active components covalently modify GSH, protein cysteines, and DNA bases, inducing stress responses and ICD. The anhydride ring of cantharidin is a strong electrophile. Upon ring opening, it covalently binds to biological amines (22). This reaction inhibits PP2A and induces ICD, while covalent binding to renal tubular epithelial proteins contributes to nephrotoxicity (23, 39). Norcantharidin retains the anhydride ring but has reduced electrophilicity, with significantly lower nephrotoxicity (29). The tissue distribution of cantharidin, with high concentrations in liver and kidney, provides a pharmacokinetic basis for its hepatotoxicity and nephrotoxicity (40).

The C9-C11 epoxide and C12-C13 unsaturated lactone ring of triptolide covalently bind to cysteine residues of the XPB subunit of RNA polymerase II, inhibiting transcription and inducing apoptosis, while also contributing to hepatotoxicity (11, 26). The enolide ring of bufadienolides is the recognition and binding site for Na^+^/K^+^-ATPase. Inhibition of this enzyme leads to intracellular Ca²^+^ overload, inducing apoptosis and ICD in tumor cells but cardiotoxicity in cardiomyocytes (27, 41). The C8 acetyl and C14 benzoyl groups of aconitine form the diester structure crucial for sodium channel activation, serving as the pharmacophore for cardiotonic effects and the source of cardiotoxicity and neurotoxicity (24, 25). Hydrolysis to monoesters or amino alcohols significantly reduces toxicity (42). The α, β-unsaturated carbonyl of yuanhuacine and ingenane diterpenoids contributes to PKC activation and gastrointestinal toxicity (43, 44).

At subtoxic concentrations, selectivity for specific immune-signaling nodes reflects GSH buffering, differences in cysteine pKa and accessibility, and cell-type-specific redox homeostasis. At high doses, GSH depletion leads to non-specific covalent modification. Chemoproteomic studies show that covalent KEAP1 inhibitors can achieve selectivity by targeting Cys151 within the BTB domain, whereas reversibly binding electrophiles do not increase KEAP1 thermostability (45, 46). Dendrobine directly targets the KEAP1 Kelch domain to disrupt KEAP1-NRF2 interaction, activating antioxidant defense (47). These findings support a dose-dependent switch from selective signaling modification to non-specific toxicity. This logic can be summarized as the “three-step model of electrophilic stress-induced ICD”: (1) covalent modification of GSH and protein cysteines; (2) stress activation with ROS accumulation, ER stress, and mitochondrial damage; and (3) release of damage-associated molecular patterns (DAMPs), including CRT exposure, high mobility group box 1 (HMGB1) release, and ATP efflux (45–47).

### 2.3 Ion-channel and pump modulators and non-electrophilic alkaloids

Matrine and oxymatrine are quinolizidine alkaloids that lack strong electrophilic groups and have relatively low toxicity. They primarily exert immunomodulatory effects by interfering with PI3K/AKT, STAT3, and NF-κB signaling (48–50). The N→O coordination bond of oxymatrine enables it to act as a mild ROS inducer at low doses and participate in epigenetic regulation. Oxymatrine downregulates PD-L1 in colorectal cancer cells by promoting DNA demethylation (28), suggesting that at low doses the N→O bond may act through epigenetic modulation rather than direct cytotoxicity. At high doses, it induces oxidative damage. In contrast, the toxicity and activity of aconitine alkaloids both originate from the diester structure (24, 25). Aconitine causes arrhythmias and fatal ventricular fibrillation by persistently activating sodium, calcium, and potassium channels in cardiomyocyte membranes, leading to calcium overload, mitochondrial dysfunction, pyroptosis, and autophagy (51, 52). Bufadienolides in toad venom produce cardiotoxicity by inhibiting Na^+^/K^+^-ATPase (27). These non-electrophilic toxins illustrate that toxicity-efficacy homology is not limited to covalent electrophiles.

### 2.4 Inorganic arsenic

ATO primarily acts as trivalent arsenic (As^III^). Its toxicity and efficacy mechanisms overlap: at low doses, it induces ER stress and mitochondrial damage, activating cGAS-STING; at high doses, it directly induces apoptosis and necrosis (14, 15, 53). ATO can also induce ferroptosis through GSH depletion and GPX4 inactivation, as shown in a nano-ultrasound contrast agent delivery system (65). Its success in APL relies on the differentiation-inducing and immune-activating effects of low-dose arsenic rather than high-dose cytotoxicity (13). Realgar (As_4_S_4_), another arsenic-containing mineral medicine, can be detoxified by water grinding, and nano-realgar improves bioavailability and antitumor activity (54, 55).

### 2.5 Boundary cases: low-toxicity immunomodulators

Epimedium flavonoids, including icariin, icaritin, and icariside I, belong to the 8-prenylflavonoid class. They lack strong electrophilic groups, have low toxicity, and exert immunomodulatory effects mainly by regulating signaling pathways, such as inhibiting IKK-α/NF-κB to downregulate PD-L1 and inducing pyroptosis or ferroptosis (18, 56–58). Scolopentide is an anti-hepatoma peptide isolated from Scolopendra subspinipes mutilans, with sequence RAQNHYCK. It binds death receptors DR4 and DR5, inducing apoptosis through FADD, caspase-8, and caspase-3 (32). These agents are included only as boundary cases because they illustrate dose-dependent immunomodulation and toxicity-efficacy separation rather than classical toxicity-efficacy homology.

### 2.6 Toxicity characteristics and dose dependence

The toxicity spectra of core toxic TCMs are broad and affect the heart, liver, kidneys, nervous system, and reproductive organs. Aconitine causes arrhythmias and fatal ventricular fibrillation through persistent ion-channel activation, calcium overload, mitochondrial dysfunction, pyroptosis, and autophagy (51, 52). Bufadienolides produce cardiotoxicity by inhibiting Na^+^/K^+^-ATPase (27). Cantharidin induces arrhythmias (59) and acute hepatotoxicity involving TNF-α, IKK-α, Bcl-2, Bax, and caspase-3 (60). Triptolide hepatotoxicity involves TLR4/NF-κB/Mincle-mediated M1 macrophage activation (57). Cantharidin causes renal tubular damage through stress response, CIDE proteins, MAPK, AMPK, and HIF-1 pathways (58). Aconitine induces neuronal apoptosis and energy metabolism dysfunction via mitochondrial calcium overload, AMPK inhibition, and ferroptosis (61, 62). Triptolide impairs mouse oocyte quality (63), and cantharidin causes testicular oxidative damage, mitophagy, and blood-testis barrier disruption (64).

For electrophilic components, these toxicities can arise from non-specific covalent modification; by contrast, ion-channel and pump modulators cause toxicity mainly through persistent functional modulation. As illustrated in **Figure 2**, three dose intervals can be conceptually described: the immunoactivation window (immune activation > toxicity), the therapeutic window (efficacy > toxicity), and the toxicity window (toxicity dominant). The immunoactivation window is a subset of the therapeutic window and should not be interpreted as a strict hormetic response without same-endpoint dose-response data.

**Figure 2:**
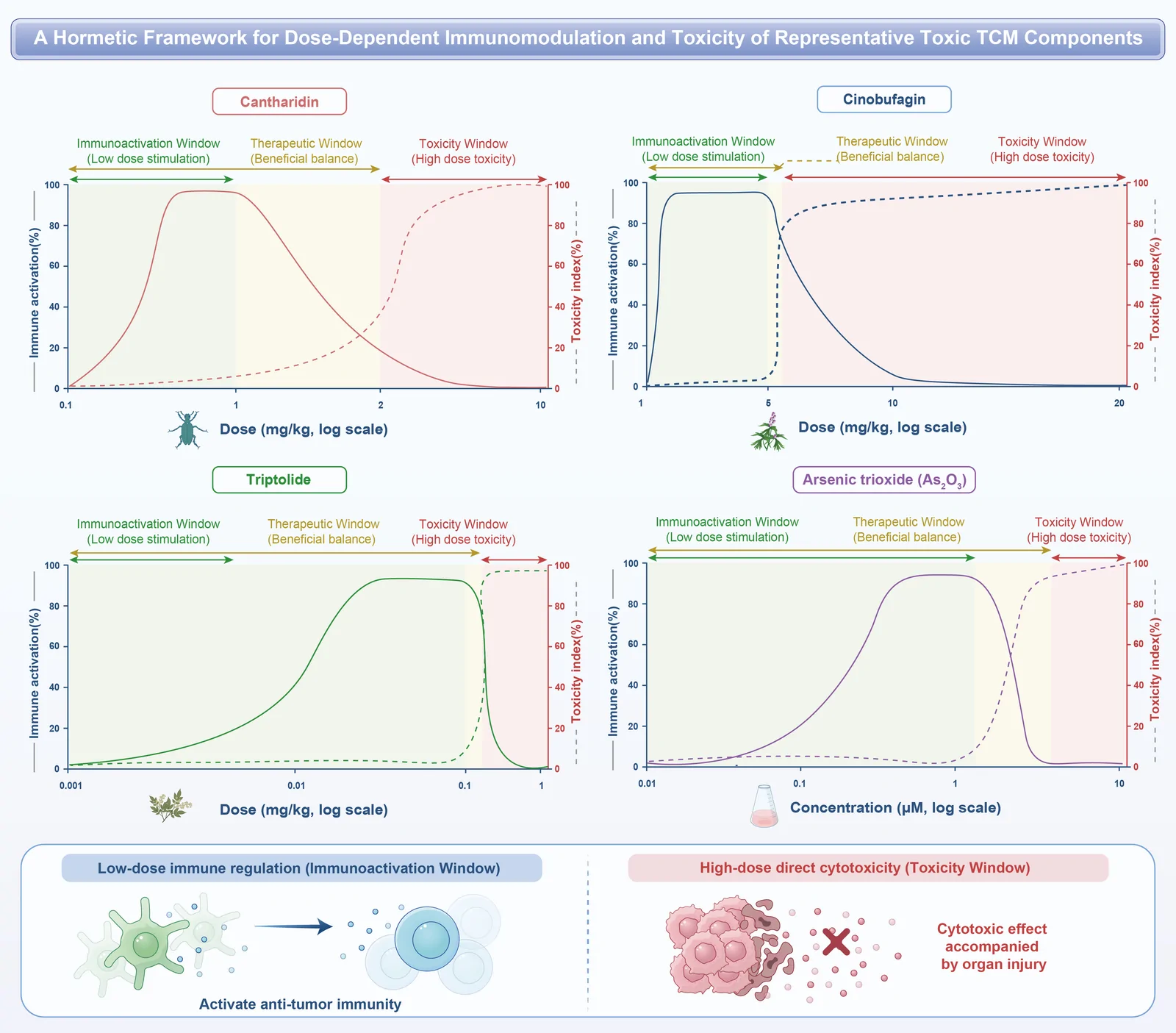
A hormetic framework for dose-dependent immunomodulation and toxicity of representative toxic TCM components. Four independent panels are shown, each with its own x-axis (log scale) and units. The green shaded area represents the operational immunoactivation window, the yellow shaded area represents the therapeutic window, and the red shaded area represents the toxicity window. The solid curve represents immune activation, and the dashed curve represents toxicity. Approximate boundaries: cantharidin, immunoactivation 0.1–1 mg/kg, therapeutic 0.1–2 mg/kg, toxicity >2 mg/kg (16, 23); cinobufagin, immunoactivation 1–5 mg/kg, therapeutic 1–6 mg/kg, toxicity >6 mg/kg (17, 37, 38, 75, 76); triptolide, immunoactivation 0.001-0.1 mg/kg, therapeutic 0.001-0.3 mg/kg, toxicity >0.3 mg/kg (11, 26); arsenic trioxide, immunoactivation 0.01-2 μM, therapeutic 0.01-5 μM, toxicity >5 μM (14, 15). The green arrow is shown within the yellow arrow to indicate that the immunoactivation window is conceptually a subset of the therapeutic window. The shaded bands are displayed as consecutive dose ranges for visual clarity.

## 3 Core mechanisms of active components from toxic TCMs in regulating the TIME

Active components of toxic TCMs remodel the TIME through multi-target, multi-pathway synergy, spanning ICD induction, TAM reprogramming, CD8^+^ T-cell activation, MDSC/Treg inhibition, checkpoint regulation, cytokine modulation, and stromal remodeling (**Figure 3**). These mechanisms and the dose-dependent bidirectional regulation that unifies them are summarized in **Table 2**. This review distinguishes direct tumor-cell effects, direct immune-cell effects, secondary immune responses, stromal/vascular effects, peripheral immunity, and local TIME remodeling. Marker-based changes are interpreted as correlative unless functional antitumor immunity has been demonstrated.

**Figure 3:**
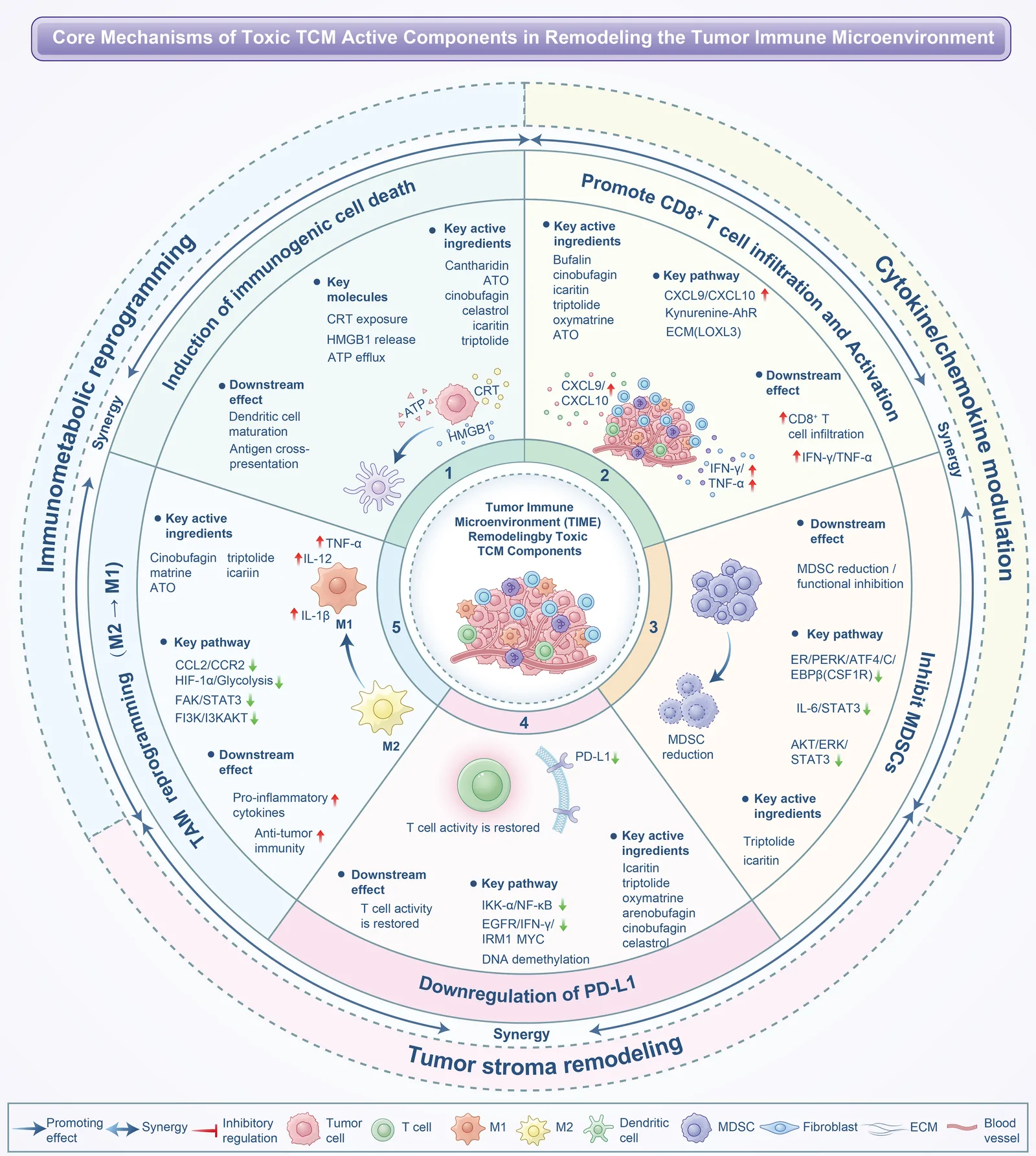
Core and auxiliary mechanisms of toxic TCM components in remodeling the tumor immune microenvironment. The figure uses a concentric or hub-and-spoke structure. The central circle is labeled “Tumor Immune Microenvironment Remodeling by Toxic TCM Components”. The inner ring summarizes five core mechanisms supported by functional evidence in at least one immunocompetent model: (i) Induction of ICD; (ii) TAM reprogramming (M2 → M1); (iii) Promote CD8^+^ T-cell infiltration/activation; (iv) Inhibit MDSCs; and (v) Downregulation of PD-L1. The outer ring summarizes three auxiliary mechanisms supported mainly by marker-level or associative evidence: (i) Immunometabolic reprogramming; (ii) Cytokine/chemokine modulation; and (iii) Tumor stroma remodeling.

**Table 2:** Summary of core mechanisms by which active components of toxic TCMs regulate the tumor immune microenvironment.

Mechanism	Key active components	Major effects	Key signaling pathways / targets	References
Induction of ICD/RCD	Cantharidin, ATO, cinobufagin, celastrol, icaritin, norcantharidin, triptolide	CRT exposure, HMGB1/ATP release, DC maturation, adaptive immunity	ROS/ER stress, cGAS-STING, PP2A, PANoptosis, autophagy, ferroptosis, pyroptosis, cuproptosis, necroptosis	(14–18, 56, 66–74)
TAM reprogramming (M2→M1)	Cinobufagin, CKI, triptolide, matrine, icariin, epimedokoreanin B, ATO	Decreased M2 markers (CD206, Arg-1), increased M1 markers (TNF-α, IL-1β)	CCL2/CCR2, HIF-1α/glycolysis, FAK/STAT3, NF-κB, PI3K/AKT, integrin β3/PPARγ	(37, 38, 75–85)
CD8^+^ T-cell infiltration/activation	Bufalin, cinobufagin, icariin, icariside I, triptolide, oxymatrine, ATO	Increased CD4^+^/CD8^+^ T-cell numbers, enhanced IFN-γ/TNF-α production	CXCL9/CXCL10, kynurenine-AhR, ECM remodeling (LOXL3), MYC/PD-L1	(17, 86–91, 105)
Treg inhibition	Oxymatrine	Reduced CD4^+^CD25^+^Foxp3^+^ Treg proportion	MYC/PD-L1, PD-L1 signaling	(91)
MDSC inhibition	Triptolide, icariin, Gansui-Banxia Decoction	Reduced MDSC frequency (especially G-MDSC), induced apoptosis	ER stress/PERK/ATF4/C/EBPβ (CSF1R), IL-6/STAT3, AKT/ERK/STAT3	(92–94)
Immune checkpoint regulation (PD-L1 downregulation)	Icaritin, triptolide, oxymatrine, arenobufagin, cinobufagin, celastrol	Decreased tumor cell PD-L1 expression, enhanced T-cell recognition	IKK-α/NF-κB, EGFR/IFN-γ/IRF1, MYC, DNA demethylation, HSP90β/STAT3, SHP2/MHC-I	(18, 28, 36, 75, 91, 95–99)
Immunometabolic reprogramming	ATO + cryptotanshinone, icariside I	Enhanced glycolysis in M1, reduced lactate, kynurenine-AhR inhibition	AMPK, HIF-1α/PI3K/AKT/mTOR, kynurenine-AhR	(85, 89)
Cytokine/chemokine network modulation	Triptolide, ATO, icariin, icariside I, cantharidin	Downregulation of IL-10, TGF-β, TNF-α, IL-6; upregulation of CXCL9, CXCL10, IFN-γ	NF-κB, kynurenine-AhR	(78, 81, 87, 89, 100, 101)
Tumor stroma remodeling	Triptolide, oxymatrine, ATO, celastrol, Fuzi	CAF inactivation, ECM softening, vascular normalization, reduced collagen	TGF-β, SPTLC2/S1P, LOXL3, miR-144/TNC	(90, 102–105, 187)

### 3.1 Induction of immunogenic cell death and regulated cell death

ICD is a mode of programmed cell death that releases DAMPs, including CRT, HMGB1, and ATP, thereby activating adaptive immunity (16). Electrophilic covalent modification triggers ROS accumulation, ER stress, and DAMP release, representing a direct mechanistic manifestation of toxicity-efficacy homology. Multiple active components induce ICD through distinct mechanisms, as summarized in **Table 3**.

**Table 3:** Comparison of characteristics of immunogenic cell death and regulated cell death induced by active components of toxic TCMs.

Active component	Tumor model (in vitro/in vivo)	ICD/RCD markers	Key pathways	Immune outcome	Combination with ICI	References
Cantharidin (nano)	4T1 breast cancer, LLC lung cancer	CRT exposure, ATP release, HMGB1 translocation	Ferroptosis, PP2A inhibition	DC maturation, CD8^+^ T-cell infiltration	Enhanced anti-PD-1 efficacy	(16)
ATO	HCC, CRC	CRT exposure, HMGB1/ATP release	ROS/ER stress, cGAS-STING	DC maturation, CD8^+^ T-cell activation	Synergistic with PD-1/PD-L1 inhibitors	(14, 15)
Cinobufagin	Glioma, lung cancer	PANoptosis (pyroptosis + apoptosis + necroptosis)	PANoptosome	M1 microglia/macrophage polarization, CD4^+^/CD8^+^ T-cell infiltration	Prolonged survival with anti-PD-1	(17)
Celastrol (nanoemulsion)	Melanoma	Strong ICD (CRT, HMGB1, ATP)	ROS, ER stress	Abscopal effect, DC activation	Distant tumor inhibition with anti-PD-1	(66)
Icaritin	HCC	Pyroptosis (GSDMD, GSDME)	Caspase-1, caspase-3	Macrophage M1 conversion, CD8^+^ T-cell infiltration	Not reported	(56)
Icariin	CRC	Ferroptosis (mitochondrial dysfunction)	IFN-γ from CD8^+^ T cells	Ferroptosis-dependent ICD	Enhanced anti-PD-1	(67)
Norcantharidin	Bladder cancer (acidic condition)	Autophagy-dependent ICD	Autophagy	Tumor-infiltrating T lymphocytes increased	Not reported	(68)
Triptolide	Cervical cancer	CRT exposure, HMGB1/ATP release	ER stress, oxidative stress	DC maturation (implied)	Not reported	(69)
Triptolide	Head and neck cancer	GSDME-mediated pyroptosis	Mitochondrial hexokinase II	Not determined in this study	Not reported	(72)
Celastrol-Cu nanoparticles	Breast cancer	Cuproptosis + ICD	GSH scavenging, DLAT aggregation	DC maturation, CD8^+^ T-cell infiltration	Enhanced anti-PD-L1	(73)
Celastrol	Gastric cancer	Necroptosis	RIP1/RIP3/MLKL	Inflammation modulation	Not reported	(74)

Cantharidin-loaded nanomedicine triggers CRT exposure, ATP release, and HMGB1 translocation, promoting DC maturation (16). In 4T1 and LLC models, it combines with PD-1 blockade to enhance CD8^+^ T-cell infiltration (16). ATO induces ICD in hepatocellular carcinoma (HCC) cells via the ROS/ER stress axis, with CRT exposure and HMGB1/ATP release (14). It also activates the cGAS-STING-IFN pathway, promoting CD8^+^ T-cell infiltration (15). In a nano-delivery system, ATO-induced ferroptosis and photothermal-induced ICD further enhanced DC maturation and CTL infiltration (65). Cinobufagin induces PANoptosis in glioma and lung cancer, increasing ICD and promoting M1-type microglia/macrophage polarization and CD4^+^/CD8^+^ T-cell infiltration (17). Celastrol nanoemulsion induces strong ICD and downregulates PD-L1, producing an abscopal effect in melanoma (66). Icaritin induces pyroptosis in HCC via caspase-1-GSDMD and caspase-3-GSDME pathways, promoting macrophage M1 conversion and CD8^+^ T-cell infiltration (56). Icariin-induced ferroptosis in a colorectal cancer (CRC) model depends on CD8^+^ T-cell-derived IFN-γ and enhances anti-PD-1 efficacy (67). Norcantharidin induces autophagy-dependent ICD in bladder cancer cells under acidic conditions and increases tumor-infiltrating T lymphocytes (68). Triptolide triggers ICD in cervical cancer cells via ER stress and oxidative stress, with CRT exposure and HMGB1/ATP release (69).

Beyond apoptosis and ICD, toxic TCM components induce multiple regulated cell death (RCD) pathways. Acevaltrate induces ferroptosis by targeting PCBP1/2 and GPX4 in CRC (70). Baohuoside I induces GSDME-dependent pyroptosis and synergizes with cisplatin in lung adenocarcinoma (71). Triptolide induces GSDME-mediated pyroptosis in head and neck cancer by suppressing mitochondrial hexokinase II (72). Celastrol-Cu nanoparticles induce self-amplified cuproptosis and ICD, augmenting cancer immunotherapy (73). Celastrol also induces necroptosis in gastric cancer cells (74). These findings indicate that the immunomodulatory effects of toxic TCMs are not limited to apoptosis and ICD but involve ferroptosis, pyroptosis, cuproptosis, and necroptosis. This broader RCD spectrum may help overcome apoptosis resistance and enhance antitumor immunity (70–74). However, for most of these pathways, functional evidence that RCD directly drives CD8-dependent antitumor immunity *in vivo* remains limited.

### 3.2 Reprogramming of TAM polarization

TAMs are abundant in the TIME, and M2 polarization is a core driver of immunosuppression. Active components of toxic TCMs can reprogram TAM polarization by interfering with key signaling pathways. Cinobufagin inhibits M2-type TAM polarization and promotes M1-type conversion across multiple cancer types. In ovarian cancer, the mechanism involves inhibiting the CCL2/CCR2 pathway (38). In lung cancer, cinobufagin reduces the M2 marker CD206 and upregulates TNF-α and IL-1β in M0/M1 macrophages (37). In non-small cell lung cancer (NSCLC), cinobufagin reverses M2 macrophage polarization by targeting HIF-1α and glycolytic metabolism (75). In triple-negative breast cancer (TNBC), cinobufagin upregulates MME, inhibiting FAK and STAT3 phosphorylation to block M2 polarization (76).

Compound Kushen Injection (CKI) activates TNFR1-mediated NF-κB and p38 MAPK signaling in TAMs, reversing immunosuppression, increasing CD8^+^ T-cell infiltration, and enhancing the efficacy of low-dose sorafenib against HCC (77). Triptolide also inhibits M2 polarization. In CRC, it downregulates tumor-derived CXCL12 via the NF-κB and ERK1/2 axis, reducing TAM infiltration (78). In breast cancer, it acts as an integrin β3 inhibitor, suppressing M2 polarization by downregulating PPARγ expression (79). In ovarian cancer, triptolide reverses M2-type TAM polarization by inhibiting the PI3K/Akt/NF-κB signaling pathway (80). Triptolide also reduces the secretion of IL-10 and TGF-β by TAMs by inhibiting NF-κB signaling (81).

Matrine inhibits the mTOR/PI3K/Akt signaling pathway, reducing the polarization of M2-type RAW264.7 macrophages induced by Lewis lung carcinoma cells and suppressing epithelial-mesenchymal transition (EMT) of lung cancer cells induced by M2-like macrophages (82). Icariin blocks the conversion of macrophages to the M2 phenotype by inhibiting PI3K/AKT signaling in a CRC model (83). Epimedokoreanin B increases M1-type macrophage infiltration and reduces M2-type and other immunosuppressive subsets in TNBC (84). ATO inhibits M2-type macrophage polarization and promotes conversion to the M1 antitumor phenotype (85). In a nano-ultrasound contrast agent system, ATO combined with photothermal therapy increased the M1/M2 ratio in the TME and promoted M1 polarization (65).

### 3.3 Regulation of T-cell function and treg inhibition

T cells are central to adaptive antitumor immunity. Toxic TCM components can promote effector T-cell activation/infiltration and inhibit Treg function via multiple pathways. Bufalin activates antitumor T-cell immune responses by driving the polarization of tumor-infiltrating macrophages from the M2 to the M1 phenotype, enhancing the antitumor activity of anti-PD-1 antibodies in HCC (86). Cinobufagin induces PANoptosis in glioblastoma, significantly increasing CD4^+^ and CD8^+^ T-cell infiltration (17). Icariin promotes CD8^+^ T-cell infiltration into tumor tissue by downregulating immunosuppressive cytokines (TNF-α, IL-10, IL-6) and upregulating chemokines (CXCL9, CXCL10) in an NSCLC model (87). In a melanoma model, icariin combined with CpG oligonucleotides increases infiltration of CD8^+^ T cells expressing IFN-γ and TNF-α (88). Icariside I downregulates nuclear PD-1 expression in cytotoxic T lymphocytes by blocking the kynurenine-AhR pathway, significantly increasing CD8^+^ T-cell numbers (89). Triptolide increases CD8^+^ T-cell infiltration and enhances anti-PD-L1 efficacy in pancreatic cancer by inhibiting pancreatic stellate cell activation and regulating LOXL3 expression to remodel the extracellular matrix (ECM) (90). Treg inhibition is achieved by multiple toxic TCM components. Oxymatrine downregulates Tregs and promotes CD4^+^ T, CD8^+^ T, and NKT cells by targeting the MYC/PD-L1 pathway (91).

### 3.4 Inhibition of MDSCs

MDSCs are a key immunosuppressive cell population in the TIME. Active components of toxic TCMs can reduce MDSC recruitment and impair their function by inhibiting signaling pathways such as STAT3 and ERK. In HCC, triptolide reduces the frequency of CSF1R^+^ MDSCs and reprograms their immunosuppressive function by inhibiting PERK phosphorylation in the ER stress pathway, blocking activation of downstream transcription factors ATF4 and C/EBPβ, thereby downregulating CSF1R expression (92). Icariin reduces MDSC generation and activation by inhibiting splenic extramedullary hematopoiesis (93). Gansui-Banxia Decoction reduces MDSC accumulation in peripheral blood and tumor tissue of tumor-bearing mice by downregulating phosphorylation of AKT, ERK, and STAT3 signaling pathways (94).

### 3.5 Regulation of immune checkpoint molecules

Active components of toxic TCMs can relieve inhibition of T-cell function by downregulating immunosuppressive checkpoint molecules. Icaritin directly targets and inhibits IKK-α, blocking NF-κB signaling activation, thereby downregulating tumor cell PD-L1 expression in a dose-dependent manner, and shows synergistic antitumor effects when combined with anti-PD-1 antibodies (18). Triptolide downregulates PD-L1 expression in NSCLC by inhibiting both the EGFR signaling pathway and the IFN-γ-JAK-STAT-IRF1 signaling axis (95). It also reverses IFN-γ-induced PD-L1 upregulation in TNBC and oral squamous cell carcinoma (OSCC) (96, 97). Oxymatrine downregulates PD-L1 expression in melanoma cells by inhibiting the MYC/PD-L1 pathway (91). It also reduces PD-L1 expression in CRC cells by promoting DNA demethylation (28), suggesting that its N→O bond may exert immunomodulatory effects via epigenetic modifications at low doses. Arenobufagin targets HSP90β, inhibiting the HSP90β-STAT3-PD-L1 signaling axis (36). Cinobufagin targets HIF-1α to reprogram glucose metabolism in NSCLC-associated macrophages, inhibiting M2-type macrophage function and reducing PD-L1 expression (75). Celastrol upregulates MHC-I molecule expression by targeting SHP2 and activating the JAK/STAT1 pathway, enhancing CD8^+^ T-cell immune recognition of melanoma (98). Arsenic induces cyclin D1 degradation and upregulates PD-L1 in esophageal squamous cell carcinoma (ESCC), sensitizing tumors to PD-1/PD-L1 checkpoint inhibitor therapy (99). Although ATO can upregulate PD-L1 on tumor cells, it shows significant synergistic effects when combined with anti-PD-1 antibodies in various tumor models via the ICD mechanism (14).

### 3.6 Immunometabolic reprogramming

Immune cell function is tightly coupled with metabolism. Active components of toxic TCMs can regulate immune cell differentiation and function by interfering with metabolic reprogramming. ATO combined with cryptotanshinone activates the AMPK signaling pathway, enhancing glucose utilization and glycolysis in macrophages to promote M1-type polarization (85). Icariside I was identified as a novel inhibitor of the kynurenine-AhR pathway. By downregulating key metabolites and enzymes in this pathway, it blocks AhR activation and reverses T-cell functional inhibition (89). These findings link toxic TCM components to immunometabolic control, but the causal contribution of these metabolic changes to antitumor immunity requires further functional validation.

### 3.7 Regulation of cytokine and chemokine networks

Multiple components suppress immunosuppressive cytokines. Triptolide significantly downregulates TNF-α, IL-6, IL-10, and TGF-β by inhibiting NF-κB activation (78, 81). Arsenic downregulates IL-10 in an acute myeloid leukemia model (100). Icariin downregulates TNF-α, IL-10, and IL-6 in NSCLC (87). Regarding promotion of immunostimulatory factors, icariin upregulates CXCL9 and CXCL10 expression in NSCLC, promoting CD8^+^ T-cell chemotaxis (87). Icariside I enhances IFN-γ secretion by blocking the kynurenine-AhR pathway (89). After cantharidin treatment, expression of inflammatory factors and immune checkpoint genes PD-1/PD-L1 is significantly reduced (101). These cytokine changes are mostly correlative, and whether they are required for antitumor efficacy remains to be determined.

### 3.8 Tumor stroma remodeling

The tumor stroma acts as a physical and biochemical barrier to immune cell infiltration. Active components of toxic TCMs can remodel the stroma to improve immune cell infiltration. In pancreatic cancer, triptolide disrupts the paracrine activation loop of cancer-associated fibroblasts (CAFs) by inhibiting tumor cell secretion of TGF-β, reducing collagen deposition and ECM stiffening (90). Regarding anti-angiogenesis and vascular normalization, triptolide inhibits proliferation, migration, adhesion, and angiogenesis of human umbilical vein endothelial cells by suppressing the SPTLC2/S1P signaling pathway (102). Celastrol nanocrystals exhibit antitumor effects by inhibiting tumor angiogenesis (103). Regarding ECM regulation, triptolide targets and inhibits tenascin C by upregulating miR-144 expression (104). In pancreatic cancer, low-dose ATO (1.0 μM) inhibits pancreatic stellate cell activation and regulates LOXL3 expression, increasing CD8^+^ T-cell infiltration and enhancing anti-PD-L1 efficacy (105). These stromal effects provide a rationale for combining toxic TCM components with ICIs, but direct functional evidence *in vivo* is still limited.

### 3.9 Dose-dependent bidirectional regulation

Seemingly contradictory reports on the same active component often reflect differences in dose. Triptolide inhibits NF-κB at low concentrations but activates it at high concentrations, low-dose arsenic promotes antitumor immunity whereas high-dose arsenic shows reduced immunogenicity, and arenobufagin exerts immunomodulation at low concentrations but direct cytotoxicity at high concentrations. These dose-dependent switches are a concrete manifestation of toxicity-efficacy homology and define the immunoactivation window, as illustrated by triptolide, where low doses promote memory T-cell differentiation while high doses induce immunosuppression and thymic atrophy (11, 26). Dose range and experimental model should therefore be specified when interpreting these mechanisms. This dose dependence can be further unified by the electrophilic stress-ICD axis. For electrophilic components, covalent modification of intracellular nucleophiles triggers ROS accumulation, ER stress, and mitochondrial damage, ultimately initiating ICD through DAMP release. At low doses, immunomodulatory effects predominate, whereas at high doses, direct cytotoxicity and immunosuppression dominate. Mapping this switch for each component is therefore essential for achieving attenuated toxicity and enhanced efficacy.

### 3.10 Spatial immune architecture and emerging evidence

Recent advances in spatial transcriptomics and multiplex imaging have begun to reveal the spatial organization of the TIME following toxic TCM intervention. A constitutive interferon-high immunophenotype defines response to immunotherapy in CRC, characterized by cytotoxic lymphocytes and antigen-presenting macrophages, with CD74 abundance predicting clinical benefit (106). In small cell lung cancer, spatial analysis identified an MT² niche consisting of antitumoral macrophages, CD8^+^ T cells, and natural killer T cells, which correlates with superior survival and predicts immunotherapy response (107). In NSCLC, spatial multi-omics identified resistance and response signatures, including proliferating tumor cells, granulocytes, and vessels in the tumor compartment, and M1/M2 macrophages and CD4 T cells in the stroma (108). Multimodal artificial intelligence has further enabled virtual multiplex immunofluorescence from H&E slides, generating a virtual population for tumor microenvironment modeling (109). These approaches provide a framework for mapping the immunoactivation window *in situ* and for identifying spatial biomarkers that predict response to toxic TCM-based combination therapy (106–109).

## 4 Preclinical research evidence system

### 4.1 *In vitro* research models

Coculture systems are widely used to analyze TIME cell interactions regulated by toxic TCM components. In a CRC coculture system, ATO inhibits M2-type TAM polarization by disrupting the Notch signaling-dependent positive feedback loop between tumor cells and TAMs. ATO inhibits M2 polarization at concentrations as low as 0.5 μM, far below its cytotoxic concentration (>5 μM) (110). In a CRC cell-macrophage coculture system, icariin blocks M2 polarization by inhibiting PI3K/AKT signaling (83). In a lung cancer cell-macrophage coculture system, matrine inhibits M2-type macrophage polarization and reduces production of IL-4, IL-10, and Arg-1 by suppressing mTOR/PI3K/Akt signaling (82).

Three-dimensional tumor spheroid and organoid models better simulate cell-cell interactions, ECM environment, and gradients of hypoxia and nutrients. Patient-derived organoids combined with organ-on-a-chip technology can be used to construct complex *in vitro* models containing tumor cells, fibroblasts, endothelial cells, and immune cells to simulate tumor-stroma interactions and evaluate drug responses (111). Organoid-immune cell coculture models more physiologically mimic tumor-immune interactions and allow prediction of immunomodulatory efficacy and screening of responsive patient populations (112). However, these advanced models have not yet been systematically applied to most toxic TCM components.

### 4.2 Animal model evidence

In syngeneic tumor models, cantharidin (1 mg/kg i.p.) increased CD4^+^/CD8^+^ T-cell ratios, reduced Tregs, and downregulated PD-1/PD-L1 in the H22 mouse liver cancer model (101). Icaritin (100 mg/kg oral) stimulated CD8^+^ T-cell infiltration by inducing pyroptosis and promoting macrophage M1 conversion (56). Triptolide (0.2 mg/kg i.p.) combined with PD-1 blockade significantly reduced tumor volume in a glioblastoma model (113). Icariin (75 mg/kg oral) promoted CD8^+^ T-cell infiltration by downregulating TNF-α, IL-10, and IL-6 and upregulating CXCL9 and CXCL10 (87). In breast cancer models, cantharidin nanomedicine induced ferroptosis and ICD in TNBC, promoting DC maturation (16). Epimedokoreanin B treatment increased infiltration of CD8^+^ T cells and M1-type macrophages while reducing immunosuppressive subsets (84). In melanoma models, icariin combined with CpG oligodeoxynucleotides prolonged survival (88). Celastrol nanoemulsion combined with anti-PD-1 significantly improved the distal tumor inhibition rate (66). Metastatic tumor models provide further evidence for the inhibitory effects of these components on tumor metastasis. In a breast cancer lung metastasis model, arsenic significantly reduced lung metastatic nodules by inducing necroptosis and ferroptosis (114). Triptonide effectively inhibited lung metastasis in a TNBC lung metastasis model by degrading Twist1 and Notch1 (115). These findings support the antitumor potential of toxic TCM components.

### 4.3 Limitations of preclinical models

Immunocompetent mouse immune systems differ significantly from humans in cytokine networks, checkpoint regulation, and metabolism. Although humanized mouse models partially compensate for species differences, they are complex and costly to construct, and the reconstituted human immune system remains functionally incomplete. Organoid models retain patient tumor heterogeneity and genetic mutation profiles but lack a complete vascular system and multi-directional interactions with immune cells. Two factors further increase the unpredictability of preclinical results. First, orally administered drugs are subject to gut microbiota metabolism. For example, icariin is hydrolyzed to icariside II and further metabolized to icaritin (116). Second, clinical application of toxic TCMs is often based on syndrome differentiation, whereas most tumor models are established under normal immune status. Preclinical studies, ranging from *in vitro* coculture models and 3D organoids to syngeneic tumor models, metastatic models, and humanized models, support the efficacy and mechanisms of active components of toxic TCMs in regulating the TIME at different levels. Single-cell and spatial omics have shifted pharmacodynamic evaluation from “bulk average” to “single-cell precision” (106–109). However, existing preclinical models still have significant limitations regarding species differences in the immune system, completeness of TIME recapitulation, and interference from microbiota metabolism. Future research should prioritize humanized patient-derived xenograft models and organoid-immune cell coculture systems, combined with TCM syndrome animal models and spatial multi-omics technologies, to bridge the translational gap between preclinical and clinical studies.

## 5 Current status and evidence from clinical research

### 5.1 Existing clinical practice

Some active components of toxic TCMs have entered clinical studies at various phases. Representative clinical evidence is summarized in **Table 4**. The success of ATO in the treatment of APL is a milestone in the modern translation of toxic TCMs. ATO targets and degrades PML-RARα, inducing APL differentiation and apoptosis. When combined with ATRA, this regimen has become a first-line standard treatment for APL (13). The success of arsenic provides a paradigmatic model for translating other toxic TCMs: defining the immunoactivation window, identifying molecular targets, and designing rational combination regimens. In APL treatment, assessment of minimal residual disease (MRD) has significant prognostic value, with blood arsenic concentration levels negatively correlated with MRD levels after induction therapy (117). Realgar-Indigo naturalis formula showed efficacy comparable to intravenous ATO in pediatric APL, with shorter hospital stays and lower infection and cardiotoxicity rates (118).

**Table 4:** Clinical research progress of active components of toxic TCMs.

Active component / preparation	Cancer type	Study type	Key efficacy findings	Safety / toxicity	References
ATO + ATRA	APL	First-line standard (multiple Phase III trials)	10-year DFS 92.5%; induces PML-RARα degradation	Well-tolerated with MRD monitoring	(13, 117)
Compound Realgar & Indigo Naturalis (oral)	Pediatric APL	Randomized controlled trial	Comparable efficacy to IV ATO; shorter hospital stay	Lower infection and cardiotoxicity	(118)
Huachansu injection + FOLFOX4	Advanced colon cancer	Adjuvant chemotherapy	Increased peripheral blood NK cells	No significant additional toxicity	(119)
Huachansu capsule	Breast cancer (adjuvant)	Meta-analysis of RCTs	Improved ORR, DCR, CD3^+^/CD4^+^ T cells	No difference in adverse events vs. chemo alone	(120)
Sodium cantharidinate + Vit B6 injection	Multiple cancers (adjuvant)	Expert consensus	Adjunct to chemotherapy	Manageable	(121)
Norcantharidin	HCC	Approved in China	Enhances sorafenib activity via IL-6/STAT3	Reduced nephrotoxicity vs. cantharidin	(29, 122)
Compound Kushen Injection (CKI)	NSCLC (adjuvant)	Meta-analysis	ORR 58.7% (chemo alone 45.2%); improved KPS	Reduced chemotherapy side effects	(123)
TCM formulas containing *Sophora flavescens*	Advanced NSCLC	Multicenter real-world	Median OS increased from 18.6 to 28.3 months	Not reported	(124)
Icaritin	Advanced HBV-related HCC	Phase II single-arm	Median OS 186 days, baseline and dynamic cytokines/immune checkpoint proteins associated with survival	No grade III/IV treatment-related AEs	(125)

Toad venom injection, combined with the FOLFOX4 chemotherapy regimen, significantly increased peripheral blood NK cell levels in patients with advanced colon cancer (119). Huachansu capsules are used as adjuvant chemotherapy in various malignancies, including lung cancer and HCC, with expert consensus recommending their use throughout the chemotherapy cycle (120). The compound injection of sodium cantharidinate and vitamin B6 has been used in China for over two decades as an adjunct to chemotherapy (121). Norcantharidin, approved for cancer treatment in China, enhances the antitumor activity of sorafenib in HCC by inhibiting the IL-6/STAT3 pathway (122). Compound Kushen Injection, as an adjunct to chemotherapy for NSCLC, has shown advantages in improving tumor response rates and Karnofsky performance status scores while reducing chemotherapy-related side effects (123). A multicenter real-world study on advanced NSCLC, using a target trial emulation design and following over 6000 patients for up to 10 years, found that TCM formulas containing *Sophora flavescens* combined with conventional therapy were associated with significantly improved 10-year overall survival (124). Icaritin has been evaluated in a phase II single-arm study in advanced HBV-related HCC, in which baseline and dynamic immune biomarkers were associated with overall survival (125).

### 5.2 Challenges in clinical trial design

Toxic TCMs have a narrow therapeutic window. The toxic dose of aconitine can be as low as 0.2 mg, with a lethal dose of only 2–5 mg (24). Traditional administration methods such as decoction struggle to ensure stable dissolution and uniform concentration of active components, leading to significant inter- and intra-individual variability in efficacy and toxicity (126). A Phase I clinical trial of a Fuzi-containing formula determined the maximum tolerated dose by measuring pharmacokinetic parameters of components such as benzoylmesaconine in plasma (127). For indication selection, priority should be given to “cold tumors” that respond poorly to existing ICIs, such as pancreatic cancer, ovarian cancer, and glioblastoma (17, 38). Using biomarkers to screen potential beneficiaries is key to achieving precision medicine. Baseline IL-6 levels for icaritin (125) and MRD monitoring for arsenic (117) provide useful strategies for patient stratification in clinical trials. Sequential administration of icariside I followed by anti-PD-1 antibodies, first using *Epimedium* components to remodel the TIME and then using ICIs, has shown significant synergistic effects in advanced tumor models (128). In the compatibility of Fuzi and licorice, licorice can induce expression of hepatic CYP450 enzymes, promoting metabolism of toxic components such as aconitine (129). For efficacy evaluation, traditional RECIST version 1.1 may not fully capture the unique efficacy patterns of immunomodulatory drugs. Immune-related response criteria such as iRECIST or imRECIST should be considered (130).

### 5.3 Efficacy and safety data

Existing clinical evidence suggests that toxic TCM preparations improve efficacy as adjuvant therapy in various malignancies. A meta-analysis of Compound Kushen Injection as adjuvant chemotherapy for NSCLC showed an objective response rate of 58.7% in the combination group compared with 45.2% in the chemotherapy-alone group (123). For Huachansu capsules, multiple randomized controlled trial meta-analyses indicate that they significantly improve objective response rate, disease control rate, and patient immune function indicators (e.g., CD3^+^ and CD4^+^ T-cell levels) as adjuvant therapy, and the adverse event profile of Huachansu injection combined with chemotherapy showed no significant difference compared with chemotherapy alone (120). In terms of safety, topical cantharidin (VP-102, 0.7%) for molluscum contagiosum showed mostly grade 1–2 local skin reactions, with a severe adverse event rate of only 3% (131). Long-term survival benefit is increasingly evident: ATO in APL achieved a 10-year disease-free survival of 92.5% (13), and a real-world NSCLC study reported increased median overall survival from 18.6 to 28.3 months following TCM formulas containing *Sophora flavescens* (124).

Clinical research evidence indicates that toxic TCM preparations show potential as adjuvant therapy to improve efficacy and reduce toxicity in various malignancies, including APL (13), HCC, lung cancer, and CRC (120–125). The success of arsenic in APL, which degrades the PML-RARα oncoprotein, re-establishes cellular differentiation, and activates antitumor immunity, provides a conceptual framework for translating other toxic TCMs into mechanism-driven immunotherapeutic agents (13). However, the overall level of existing clinical evidence is relatively low (120, 123). Future well-designed clinical trials incorporating patient stratification using biomarkers and optimizing combination regimens, especially sequential administration timing, are needed to obtain robust evidence for clinical decision-making and promote the transition of toxic TCMs from empirical adjuvant therapies to precision immunotherapeutic agents.

## 6 Clinical translation challenges and innovative strategies

### 6.1 Analysis of core challenges

The clinical translation of toxic TCM components faces multiple interrelated obstacles—a narrow therapeutic window, low bioavailability, and lack of quality-control (QC) standards—which form a vicious cycle: a narrow therapeutic window requires more precise dose control, but batch-to-batch component fluctuations make precise dosing difficult, and individual variability in bioavailability further amplifies toxicity risks (126).

#### 6.1.1 Challenge of toxicity control

Toxic TCMs have a narrow therapeutic window, as exemplified by aconitine, where the lethal dose is merely 2–5 mg (24). Cantharidin poisoning causes severe digestive and urinary symptoms (132, 133). A clinical case report described a 2-year-old boy who ingested a cantharidin-containing herbal remedy. Although cantharidin was undetectable in his blood 20 hours after admission, poisoning symptoms persisted for about 5 days (134). Triptolide can cause hepatotoxicity, nephrotoxicity, reproductive toxicity, and cardiotoxicity (11, 26). Its hepatotoxicity mechanism involves inducing M1 polarization of macrophages via activation of the TLR4/NF-κB/Mincle pathway (57). For electrophilic components, these toxicities can result from non-specific covalent modification of active groups in normal tissues. Long-term administration of triptolide may also lead to thymic atrophy and excessive immunosuppression, increasing the risk of opportunistic infections (11). The cardiotoxicity and neurotoxicity of bufadienolides are major obstacles limiting their systemic application (27). Therefore, the core strategy for toxicity control should focus on modulating the reactivity of toxicity-efficacy groups, limiting their distribution in non-target tissues, or accelerating their metabolic clearance from normal organs. For example, ellagic acid protects against ATO-induced cardiotoxicity in rats (135), and Shengxian Decoction ameliorates ATO-induced cardiac toxicity via AMPK/SIRT1/PGC-1α (136). LBP-4 attenuates triptolide-induced male reproductive toxicity via Nrf2/Keap1-mediated antioxidant response (137).

#### 6.1.2 Formulation and administration obstacles

Most active components of toxic TCMs have poor water solubility and extremely low oral bioavailability. The oral bioavailability of yuanhuacine in rats is only 1.14% (138). Orally administered icariin is rapidly metabolized by hydrolases in the gut microbiota into icariside II, which can be further metabolized to icaritin (116). Due to its high lipophilicity, the pharmacokinetic behavior and tissue distribution of cantharidin *in vivo* are significantly affected. After oral administration, it mainly accumulates in the liver and kidneys, which is related to its strong irritancy and low bioavailability (40). Although norcantharidin has reduced toxicity, it still suffers from poor solubility, short half-life (only about 2.5 hours), rapid metabolism, and high intravenous irritancy (29). Triptolide undergoes extensive phase I and II metabolism *in vivo*, generating various metabolites. Some metabolites may have stronger immunosuppressive activity than the parent drug, but data on their long-term exposure levels and tissue accumulation characteristics remain insufficient (11, 26).

A substantial translational gap exists between *in vitro* IC50 metrics and the *in vivo* immunoactivation window, and the factors include Cmax/AUC, protein binding, tissue distribution, hepatic metabolism, and gut microbiota metabolism. For example, Fuzi compatibility with licorice induces hepatic CYP3A enzymes, promoting aconitine metabolism and reducing exposure by about 40% (129). Therefore, defining the immunoactivation window requires integration of *in vitro* potency, *in vivo* exposure, and tissue-specific toxicity (33–35, 126).

#### 6.1.3 Standardization and quality control challenges

As natural products, the chemical complexity and batch-to-batch variability of toxic TCMs pose fundamental difficulties for QC. Toad venom from different species shows significant variability in active component content. The total content of five major bufadienolides in samples from *Bufo bufo gargarizans* was 8.15-15.93%, while in samples from *Bufo melanostictus* it was only 2.45-4.14% (139). The composition and proportion of alkaloids in Fuzi also vary significantly depending on origin and batch (140, 141). After vinegar processing of *Euphorbia kansui*, the content of 3-O-(2′E, 4′Z-decadienoyl)-20-O-acetylgenol decreased by 19.14%, while the content of ingenol increased by 92.31%, indicating drastic chemical changes directly related to the balance of toxicity and efficacy (142). The secondary metabolite profile in the roots of *Sophora flavescens* varies significantly due to ecological factors. Clear regional genetic differences exist among germplasm resources from 9 different provinces in China (143). Realgar, a traditional mineral TCM, primarily consists of As_4_S_4_, but its content and impurity profile vary significantly depending on the ore source, directly affecting efficacy and toxicity (54). *Datura metel* L. seeds contain toxic alkaloids like atropine and scopolamine. Their unprocessed extract shows clear neurotoxicity and hepatotoxicity after 28 days of repeated dosing. After traditional “Shodhana” processing, the NOAEL increased from 25 mg/kg to 100 mg/kg (144). This chemical instability makes traditional QC models based on one or a few indicator components insufficient to fully reflect the intrinsic quality, efficacy, and safety of the herb. Therefore, establishing an operational basis and inclusion criteria for toxic TCMs is essential for QC and safe application (19–21).

### 6.2 Innovative solutions

To address the three challenges mentioned above, researchers have developed multiple strategies and solution pathways. The common logical core of these strategies is: while preserving or enhancing the immunological activity of the toxicity-efficacy homology groups, reduce their non-specific exposure and toxicity in normal organs through chemical modification, physical encapsulation, metabolic regulation, and quality control. **Table 5** summarizes representative examples of each strategy.

**Table 5:** Core challenges and innovative solutions for clinical translation of toxic TCMs.

Challenge category	Specific problem	Examples	Innovative solution	Representative strategy	References
Narrow therapeutic window	Extremely narrow dose range between efficacy and toxicity	Aconitine: toxic dose 0.2 mg, lethal 2-5 mg; cantharidin: nephrotoxicity at >2 mg/kg in mice	Structural modification & prodrug design	Norcantharidin (demethylation), DCZ5418 (TRIP13 inhibitor), CCTP (triptolide-chitosan), TP-Trp (ATB^0+^-targeted), LLDT-8	(24, 26, 29, 145–147)
Poor bioavailability & solubility	Low oral bioavailability, rapid metabolism, high lipophilicity	Yuanhuacine oral bioavailability 1.14%; triptolide extensive metabolism	Advanced delivery systems (nano, liposomes, micelles, biomimetic)	mPEG-PLGA micelles, RGD/dextran sulfate nanoparticles, PEG-grafted mesoporous silica, pH-sensitive liposomes, platelet membrane-camouflaged nanoparticles, nano-realgar	(40, 138, 158–167)
Batch-to-batch variability & lack of QC standards	Chemical composition varies by species, origin, processing	Toad venom: bufadienolides 8-15% vs. 2-4%; Fuzi alkaloids vary by origin; realgar impurity profile	Q-markers, multi-component fingerprinting, whole-process QC	Arenobufagin as Q-marker; peptide marker NNAYDINEER; neoline/fuziline/songorine for Fuzi; kurarinone/norkurarinone for *Sophora*; XRD for realgar	(54, 139–144, 168–172)
Toxicity due to non-specific covalent modification	Electrophilic groups modify normal tissue proteins	Triptolide hepatotoxicity via TLR4/NF-κB/Mincle; cantharidin renal tubular damage	Detoxification via compatibility & processing	Ginsenosides (Rb1, Rg1) antagonize aconitine cardiotoxicity; *Glycyrrhiza* induces CYP3A; vinegar processing of kansui; steaming/sand-frying of Fuzi; water-grinding of realgar	(42, 57, 129, 142, 151–157)

#### 6.2.1 Structural modification and prodrug design

Structural modification and prodrug design represent the most direct chemical toxicity-reduction strategy by blocking or modifying toxicity-efficacy homology groups, preserving or enhancing the immunologically active groups, while reducing electrophilicity to minimize non-specific covalent binding. Norcantharidin, a demethylated derivative of cantharidin, has similar antitumor activity but significantly lower toxicity (29). Further structural optimization led to DCZ5418, a derivative synthesized based on the structural similarity between the aliphatic inner ring part of cantharidin and the TRIP13 inhibitor DCZ0415. DCZ5418 showed good anti-multiple myeloma activity *in vitro* and *in vivo*, with significantly lower toxicity to normal cells (145). Prodrug design for triptolide has also advanced. The prodrug CCTP, formed by conjugating triptolide with carboxymethyl chitosan, showed comparable antitumor efficacy to the parent drug in a pancreatic cancer xenograft model after oral administration, but with significantly reduced subacute toxicity (146). An amino acid-conjugated triptolide prodrug (TP-Trp), targeting the ATB^0+^ transporter highly expressed in pancreatic cancer cells, showed 5.2-fold higher uptake in pancreatic cancer cells compared to normal cells (147). LLDT-8 ((5R)-5-hydroxytriptolide), a semi-synthetic derivative of triptolide, retains the core skeleton but introduces specific functional groups, significantly reducing hepatotoxicity while maintaining antiproliferative and pro-apoptotic activities (11). Chemical modification of the steroid nucleus of bufadienolides, by introducing lipophilic groups or altering the sugar moiety, can significantly change their binding mode to Na^+^/K^+^-ATPase, reducing cardiotoxicity (27). The immunoprotective effect of *Aconitum* polysaccharides demonstrates the chemical feasibility of toxicity-efficacy separation within the same herb (30). Recent advances in PROTAC technology have further expanded this field. Icaritin-based PROTACs identified Bax inhibitor-1 as a critical target in Burkitt lymphoma (148). Rhein-based PROTACs degrade VEGFR-2 and enhance antitumor activity (149). Hederagenin-based PROTACs degrade SKP2 and suppress leukemia cell proliferation (150). These strategies provide new chemical tools for attenuated toxicity and enhanced efficacy (148–150).

#### 6.2.2 Detoxification via compatibility and processing

The principles of “mutual restraint” and “mutual detoxification” in TCM compatibility provide strategic resources for reducing the toxicity of TCMs. In the compatibility of ginseng and Fuzi, ginsenosides Rb1 and Rg1 effectively antagonize aconitine-induced cardiotoxicity by regulating calcium homeostasis in cardiomyocytes, inhibiting apoptosis and pyroptosis, and modulating sodium and potassium ion channels (151, 152). In the compatibility of licorice and Fuzi, licorice induces expression of hepatic CYP3A enzymes, promoting metabolism of toxic components like aconitine and reducing their *in vivo* exposure by about 40% (129). Glycyrrhizin combined with *Tripterygium* glycosides alleviates the nephrotoxicity of *Tripterygium*, involving inhibition of the RhoA/ROCK1 signaling pathway (153). Processing *Tripterygium* with Rehmanniae Radix Praeparata juice can reduce its reproductive toxicity (154).

Processing for toxicity reduction is a traditional TCM practice. Its chemical essence involves converting highly toxic parent glycosides/esters into less toxic secondary glycosides/acids through hydrolysis, isomerization, oxidation, and other reactions. During vinegar processing of *Euphorbia kansui*, the more toxic 3-O-(2′E, 4′Z-decadienoyl)-20-O-acetylenginol is partially converted to the less toxic ingenol, reducing systemic toxicity while retaining the diuretic effect (142). During Fuzi processing, diester aconitine is first converted by heating hydrolysis to monoester benzoylaconine, with an approximately 100-fold reduction in toxicity, and further hydrolysis yields amino alcohol aconine, the least toxic form (42). Studies show that steaming for 4.0 hours is a suitable time point for reducing Fuzi toxicity while retaining efficacy (155). Sand-frying processing converts aconitine to pyraconitine and 16-epi-pyraconitine, the latter not only having lower cardiotoxicity but even exhibiting stronger antiarrhythmic activity than propafenone and lidocaine (156). Water-grinding processing of realgar effectively reduces its hepatotoxicity, possibly related to changes in the valence state and form of arsenic (157). These processing and compatibility strategies provide practical detoxification approaches (135–137).

#### 6.2.3 Advanced delivery systems

Nano-delivery systems provide powerful tools to overcome the low bioavailability and poor targeting of active components from toxic TCMs. For passive targeting, encapsulating cantharidin in mPEG-PLGA micelles prolongs its circulation half-life, increases tumor accumulation via the EPR effect, and reduces kidney distribution (158). Dual-targeting nanoparticles loaded with triptolide (via αvβ3 integrin-RGD and scavenger receptor A-dextran sulfate interactions) induce apoptosis of both tumor cells and M2-like TAMs in a pancreatic cancer model (159). Encapsulating benzoylaconitine in biodegradable mPEG-PLGA nanoparticles not only improves its bioavailability but also exhibits significant anti-inflammatory activity by modulating NF-κB signaling (160). Encapsulating ATO in PEG-grafted mesoporous silica nanoparticles achieves sustained release in the TIME, prolonging drug action time while significantly reducing peak blood concentrations, thereby decreasing toxic exposure to normal organs like the heart and liver (161). A nano-ultrasound contrast agent (ATO/PFH NPs@Au-cRGD) has also been developed for ATO and PFH co-delivery, achieving ultrasound imaging, ferroptosis induction, and enhanced anti-PD-L1 therapy in orthotopic liver cancer (65). For active targeting, strategies such as folate receptor-targeted liposomes, RGD peptide-modified nanoparticles, and hyaluronic acid-mPEG-modified nanostructured lipid carriers have been applied for the delivery of cantharidin and norcantharidin (162, 163). CXCR4-guided liposomes combined with sorafenib and CSF1/CSF1R inhibitors have shown efficacy in overcoming drug resistance in HCC using patient-derived xenograft models (164).

Intelligent responsive delivery systems represent a more precise drug release strategy. pH-responsive nanoparticles trigger drug release under the acidic conditions of the TIME (161). Acetalated dextran nanoparticles co-encapsulating piplartine and paclitaxel exemplify the advantages of pH-responsive delivery, remaining stable at physiological pH while releasing their cargo under acidic conditions to produce synergistic cytotoxicity against breast cancer cells (165). Cell membrane biomimetic delivery has emerged as a promising approach. Platelet membrane-camouflaged matrine nanoparticles mimic platelet characteristics to actively target inflamed lung tissue (166). Cancer cell membrane-coated nanoparticles enhance tumor cell uptake via homotypic targeting. TCM-derived nanovesicles, due to their good biocompatibility and targeting ability, can serve as ideal carriers for precisely delivering active components or immunomodulatory factors from toxic TCMs to the tumor site (166). Nano-realgar (prepared by grinding or specific solvents) has nanometer-scale particles, increased surface area, and altered physicochemical/biological activity, showing significantly better efficacy in inducing apoptosis in breast cancer cells compared to crude realgar (167).

#### 6.2.4 Standardization and quality control innovation

Q-markers are shifting QC of toxic TCMs from empirical identification toward precise quantification. Based on spectrum-effect relationship analysis, key antitumor active components like arenobufagin can be selected from toad venom as quality markers (Q-markers) (168). Using the peptide marker NNAYDINEER to quantify toad venom content allows simultaneous identification and quantification of adulterants like toad skin, meat, and bone (169). Components such as neoline, fuziline, songorine, and 10-hydroxy mesaconitine may be more specific indicators of efficacy/toxicity than existing markers, providing a new basis for QC and safe application of Fuzi (140, 170). Multi-component fingerprinting technology can more comprehensively reveal the chemical composition characteristics of the herb. A UHPLC-Q-TOF/MS study identified 37 chemical constituents, including 13 alkaloids and 24 flavonoids, in Sophora flavescens (171). Tandem HPLC fingerprinting, combining HILIC and ISC separation mechanisms, constructs tandem signal fingerprints and tandem data fingerprints, enabling a more comprehensive and authentic revelation of the distribution and characteristics of the complex alkaloid components in *Sophora flavescens* (172). The establishment of XRD Fourier fingerprinting for realgar decoction pieces provides a new means for QC of mineral medicines (54). The establishment of a whole-process QC system covering the source of the raw herb, extraction process, and QC standards is the ultimate guarantee for ensuring the batch-to-batch stability and reproducibility of active components (19–21).

These challenges, including narrow therapeutic window, low bioavailability, and lack of QC standards, form a mutually reinforcing cycle. Breaking this cycle requires multi-strategy synergy, in which structural modification and prodrug design modulate the reactivity of toxicity-efficacy groups, compatibility and processing exploit natural chemical transformations, advanced delivery systems enable targeted release, and QC innovation ensures batch-to-batch stability.

## 7 Combination therapy strategies

Combining active components of toxic TCMs with existing tumor therapies is an important strategy for overcoming monotherapy limitations. Preclinical studies indicate that synergy with ICIs, chemotherapy, radiotherapy, and CAR-T therapy involves ICD induction, TAM reprogramming, CD8^+^ T-cell infiltration, PD-L1 downregulation, MDSC/Treg inhibition, EMT reversal, and vascular normalization. From the perspectives of toxicity-efficacy homology and the immunoactivation window, these combinations exploit low-dose immunomodulatory activity rather than direct cytotoxicity to precondition the TIME (**Figure 4**).

**Figure 4:**
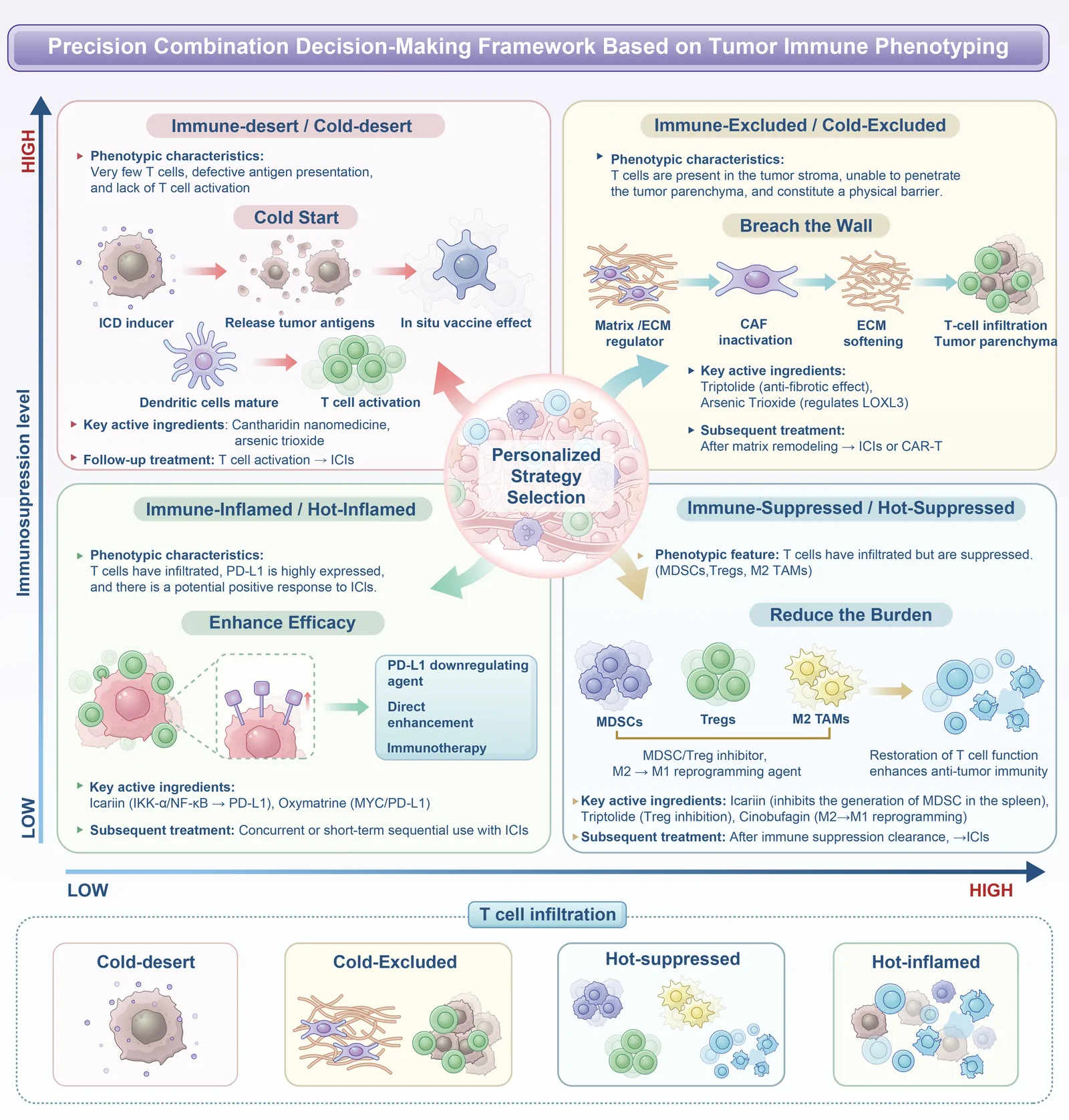
Synergistic mechanism network of active components of toxic TCMs with immune checkpoint inhibitors, chemotherapy, radiotherapy, and CAR-T therapy. This schematic illustrates the synergistic interactions between toxic TCM components and conventional cancer therapies. Shared mechanisms include ICD induction, TAM reprogramming, CD8^+^ T-cell infiltration, PD-L1 downregulation, MDSC/Treg inhibition, EMT reversal, and vascular normalization. The figure also highlights the “precondition then block” sequential strategy, in which low-dose toxic TCMs are administered first to remodel the TIME, followed by ICIs or other therapies.

### 7.1 Synergy with ICIs

Toxic TCM components remodel the TIME to create favorable conditions for ICIs. In particular, ICD-inducing agents show potent synergy with ICIs. **Table 6** summarizes the preclinical evidence for representative combination regimens. Cantharidin nanomedicine, by inducing ICD, significantly enhanced the efficacy of PD-1 blockade in 4T1 and LLC models, with the combination group showing reduced tumor volume (16). Cinobufagin induced PANoptosis in glioblastoma, producing significant synergy with anti-PD-1 and prolonging median survival (17). ATO induced ICD via the ROS/ERS pathway, showing synergy with PD-1 inhibitors in HCC (14). In an orthotopic liver cancer model, ATO nano-delivery combined with photothermal therapy and anti-PD-L1 achieved an *in situ* tumor inhibition rate of 82.5% and suppressed lung metastasis (65). Celastrol nanoemulsion, by inducing ICD and downregulating PD-L1, produced a significant abscopal effect in melanoma, and when combined with anti-PD-1, the distal tumor inhibition rate was significantly improved (66).

**Table 6:** Preclinical evidence of combination therapy with active components of toxic TCMs and immune checkpoint inhibitors or CAR-T therapy.

Active component	Tumor model	ICI/CAR-T type	Combination regimen (timing)	Key outcome	Mechanisms	References
Cantharidin nanomedicine	4T1 breast, LLC lung	anti-PD-1	Concurrent (ICD inducer + ICI)	Reduced tumor volume, increased CD8^+^ T-cell infiltration	ICD induction, DC maturation	(16)
Cinobufagin	Glioma	anti-PD-1	Concurrent	Prolonged median survival	PANoptosis, CD4^+^/CD8^+^ T-cell infiltration	(17)
Bufalin	HCC	anti-PD-1	Concurrent	Enhanced antitumor activity	TAM M2→M1 polarization	(86)
Icaritin	HCC	anti-PD-1	Concurrent	Tumor inhibition	Direct IKK-α targeting, PD-L1 downregulation	(18)
Icariside I	Gastrointestinal cancer	anti-PD-1	Sequential (precondition → ICI)	Tumor inhibition rate 76.8% (vs. concurrent ~55%)	TRPV4 targeting, cGAS-STING-IFN-I pathway	(128)
Icariin	CRC	anti-PD-1	Concurrent	Enhanced anti-PD-1 efficacy	Ferroptosis dependent on CD8^+^ T-cell-derived IFN-γ	(67)
Celastrol nanoemulsion	Melanoma	anti-PD-1	Concurrent	Distant tumor inhibition (abscopal effect)	ICD + PD-L1 downregulation	(66)
Celastrol liposomes	TNBC	anti-PD-L1	Concurrent	Remodeled TIME driven by cancer-associated adipocytes	Immunosuppressive microenvironment reprogramming	(173)
ATO	HCC	anti-PD-1	Concurrent	Synergistic effect	ROS/ERS-mediated ICD	(14)
ATO	ESCC	anti-PD-1/PD-L1	Concurrent	Sensitized tumors to ICI	Cyclin D1 degradation + PD-L1 upregulation	(99)
Oxymatrine	Melanoma, lung adenocarcinoma	anti-PD-1 / anti-PD-L1	Concurrent	Synergistic antitumor effect	MYC/PD-L1 pathway, DNA demethylation, CD8^+^ T-cell infiltration	(28, 91, 174)
Low-dose ATO	Pancreatic cancer	anti-PD-L1	Concurrent	Enhanced ICI efficacy	LOXL3 downregulation, ECM remodeling, CD8^+^ T-cell infiltration	(105)
Berbamine	CAR-T (in vitro culture)	CAR-T	Concurrent	Enhanced CAR-T persistence	Stem cell memory T-cell phenotype	(188)

Icaritin directly targeted and inhibited IKK-α, downregulating PD-L1 and showing synergy with anti-PD-1 in HCC (18). Icariside I, administered sequentially, showed significant synergy with anti-PD-1 in gastrointestinal cancers by targeting TRPV4 and activating the cGAS-STING-IFN-I pathway (128). Oxymatrine downregulated PD-L1 by inhibiting the MYC/PD-L1 pathway and promoting DNA demethylation, showing synergistic effects with anti-PD-1 in melanoma (28, 91). Additionally, bufalin enhanced anti-PD-1 activity in HCC by driving TAM polarization towards the M1 phenotype (86), and icariin enhanced anti-PD-1 efficacy by inducing ferroptosis dependent on CD8^+^ T-cell-derived IFN-γ (67). Celastrol-loaded liposomes remodeled the immunosuppressive TIME driven by cancer-associated adipocytes, achieving significant efficacy when combined with anti-PD-L1 in TNBC (173). In ESCC, arsenic induced cyclin D1 degradation and PD-L1 upregulation, sensitizing tumors to ICI therapy (99). Systems pharmacology analysis indicated that oxymatrine enhances CD8^+^ T-cell infiltration in the TIME, with significant efficacy when combined with anti-PD-L1 in a lung adenocarcinoma mouse model (174).

Preclinical studies on compound formulas containing Fuzi, such as Sini Decoction, have demonstrated enhanced antitumor immune responses in colon cancer models, with mechanisms involving upregulation of IL-2 and IFN-γ, downregulation of IL-10 and TGF-β, and increased CD8^+^ T-cell infiltration (175). However, systematic studies on *Aconitum* monomers combined with ICIs are still lacking, representing an important direction for future research. For *Genkwa Flos*, the active component yuanhuacine has been shown to activate PKC signaling and promote immunogenic cytokine expression profiles in TNBC (43), providing a theoretical basis for combination with ICIs. However, direct preclinical evidence of yuanhuacine-ICI synergy is still needed. For *Euphorbia kansui*, Gansui-Banxia Decoction has been shown to inhibit MDSC accumulation via AKT/STAT3/ERK pathways (94), suggesting a potential role in overcoming ICI resistance. For Aconitum monomers, yuanhuacine, and purified kansui components, direct evidence for ICI synergy is still lacking and represents an important direction for future research.

### 7.2 Synergy with chemotherapy

Triptolide chemosensitizes taxol-resistant lung adenocarcinoma cells by inhibiting p70S6K/GSK3/β-catenin (176). Cinobufagin enhanced the sensitivity of cisplatin-resistant lung cancer cells to cisplatin by inhibiting the PI3K/AKT and MAPK/ERK signaling pathways (177). Reversal of multidrug resistance is another important synergistic mechanism. Matrine reduced P-gp expression levels in CRC multidrug-resistant cells, increasing the intracellular accumulation of doxorubicin (178). Ingenane-type diterpenoids from *Euphorbia kansui* showed stronger multidrug resistance reversal activity in HepG-2/Adr resistant cells compared to the positive control verapamil (44). Kanesulone C, a jatrophane-type diterpenoid isolated from *Euphorbia kansui*, enhanced the efficacy of doxorubicin against resistant human breast cancer cells by approximately 85-fold (179). For reducing chemotherapy side effects, Qishen Granule (containing Fuzi) ameliorates doxorubicin-induced cardiotoxicity by upregulating SIRT3 and restoring acetylation homeostasis, with Nrf2-mediated antioxidant and anti-ferroptosis effects also contributing to its cardioprotection (180). Fuzi neutral polysaccharide effectively alleviated cyclophosphamide-induced immunosuppression in H22 tumor-bearing mice, restoring spleen and thymus indices to near-normal levels (30). Aidi Injection (containing cantharidin) can reduce chemotherapy-induced hepatorenal toxicity (181).

### 7.3 Synergy with radiotherapy

Triptolide significantly reduced the survival fraction of irradiated human glioma U251 cells by inhibiting the PI3K/Akt signaling pathway (182). Kushenol A enhanced the radiosensitivity of NSCLC cells by targeting and inhibiting IDO1 (183). Oxymatrine reversed EMT and restored the radiosensitivity of tumor cells by inhibiting the DcR3/AKT/GSK-3β signaling pathway (184). For improving tumor vascular normalization, NGR peptide-targeted liposomes loaded with triptolide induced tumor vascular normalization, increasing pericyte coverage of tumor blood vessels, decreasing vascular permeability, and elevating oxygen partial pressure in tumor tissue. When combined with radiotherapy, the tumor inhibition rate was significantly improved (185). Regarding inducing ICD to enhance the abscopal effect, celastrol combined with the chemotherapy drug SN38 in a hypoxia-sensitive nanosystem effectively induced ICD, and when combined with anti-PD-L1 antibodies, the distal tumor inhibition rate was significantly increased (186). Fuzi combined with radiotherapy has also demonstrated enhanced antitumor efficacy. In a Lewis lung cancer model, Fuzi enhanced the antitumor efficacy of radiotherapy by reducing Treg cell proportions and downregulating PD-L1 expression, thereby improving the immune microenvironment (187).

### 7.4 Potential for combination with CAR-T therapy

Toxic TCMs and their active components show potential in remodeling the TIME and enhancing CAR-T cell function. Berbamine, an alkaloid extracted from *Phellodendron amurense*, promotes a stem cell memory T-cell phenotype and enhances CAR-T persistence (188). However, direct preclinical and clinical evidence for combining toxic TCMs with CAR-T therapy remains insufficient. Future studies should focus on sequential administration, optimal dosing, and spatial immune phenotyping to guide combination regimens. The rationale is that low-dose toxic TCM components may reduce CAR-T exhaustion, remodel stroma, and improve T-cell infiltration, but dedicated studies are urgently needed.

### 7.5 Timing strategies for combination therapy and precision matching framework

Preclinical studies suggest that the optimal sequence is to administer low-dose toxic TCM first to remodel the TIME, and then to apply ICIs to relieve immunosuppression. The sequential administration of icariside I followed by anti-PD-1 antibodies achieved a tumor inhibition rate (76.8%) significantly superior to concurrent administration (~55%) or reverse sequential administration (~40%) (128). The scientific essence of this “precondition then block” strategy lies in fully utilizing the immunoactivation window of the toxicity-efficacy group at low doses: first using subtoxic doses to trigger ICD and T-cell infiltration, then using ICIs to block immunosuppressive signals, achieving temporal synergy between toxicity-efficacy and immunity.

Based on these considerations, a precision combination decision framework based on tumor immune phenotyping is proposed (**Figure 5**). This framework classifies the TIME into four phenotypes: immune-desert (cold-desert), immune-excluded (cold-excluded), immune-suppressed (hot-suppressed), and immune-inflamed (hot-inflamed). For immune-desert tumors with few T cells, the core strategy is “cold start”: using cantharidin or arsenic to induce ICD and release tumor antigens, acting as an *in situ* vaccine (14, 16), followed by ICIs after the immune system is activated. For immune-excluded tumors, where T cells exist but are trapped in the stroma, the core strategy is “breach the wall”: using triptolide or arsenic for anti-fibrosis and ECM remodeling to open channels for T-cell infiltration (90, 105), followed by ICIs or CAR-T therapy after the stromal barrier is removed. For immune-suppressed tumors, where T cells have infiltrated but are inhibited by MDSCs/Tregs/TAMs, the core strategy is “reduce the burden”: using icariin to inhibit splenic MDSC generation (93), oxymatrine to inhibit Tregs (91), or cinobufagin to drive M2→M1 reprogramming (37, 75), followed by ICIs after immunosuppressive factors are cleared. For immune-inflamed tumors, where T cells have infiltrated but PD-L1 is highly expressed, the core strategy is “enhance efficacy”: using icaritin or oxymatrine to downregulate PD-L1 expression (18, 28, 91), administered concurrently or with a short interval before/after ICIs to directly enhance immunotherapy efficacy. This framework systematically integrates tumor immune phenotype, mechanism of action of toxic TCMs, and combination timing strategies, offering a potential roadmap to guide clinical protocol design (106–109).

**Figure 5:**
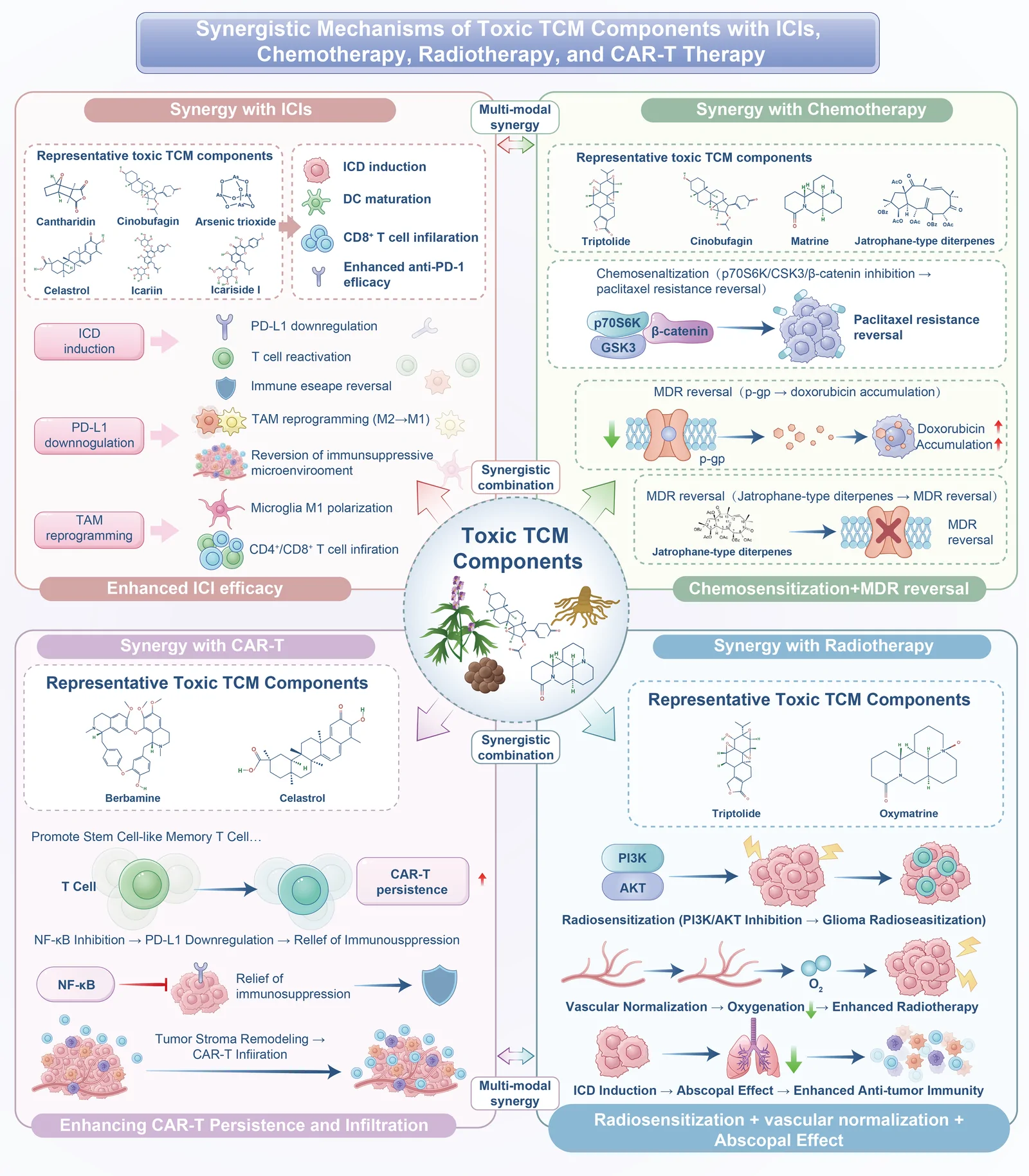
Precision combination decision-making framework based on tumor immune phenotyping. Tumors are classified into four immune phenotypes: immune-desert (cold-desert), immune-excluded (cold-excluded), immune-suppressed (hot-suppressed), and immune-inflamed (hot-inflamed). For each phenotype, a tailored combination strategy using specific toxic TCM components is proposed: “cold start” (ICD inducers such as cantharidin or arsenic), “breach the wall” (stroma/ECM modulators such as triptolide or arsenic), “reduce the burden” (MDSC/Treg inhibitors and M2→M1 reprogrammers such as icariin, oxymatrine, or cinobufagin), and “enhance efficacy” (PD-L1 downregulators such as icaritin or oxymatrine). The framework provides a roadmap for personalized combination immunotherapy guided by spatial and functional immune profiling.

In summary, active components of toxic TCMs show synergistic effects with ICIs, chemotherapy, radiotherapy, and CAR-T therapy through ICD induction, TAM reprogramming, CD8^+^ T-cell infiltration, PD-L1 downregulation, MDSC/Treg inhibition, EMT reversal, and vascular normalization. The “precondition then block” sequential strategy and the immune phenotype-guided framework provide complementary temporal and population-level approaches for clinical translation, although CAR-T combination remains in early exploration (188).

## 8 Future research directions and development opportunities

### 8.1 In-depth mechanistic analysis based on multi-omics and systems biology

Single-cell RNA sequencing (scRNA-seq) and spatial transcriptomics provide powerful tools to map TIME changes at single-cell resolution following toxic TCM intervention. scRNA-seq has revealed TIME heterogeneity in neuroblastoma and ovarian cancer (189, 190), and spatial technologies such as CODEX, spatial transcriptomics, and imaging mass cytometry have mapped immune niches, including the MT² niche in small cell lung cancer and the IFN-high immunophenotype in CRC (106–109). These approaches support single-cell precision in dissecting the multi-target regulation by toxic TCMs.

Systems pharmacology and multi-omics enable analysis of complex regulatory mechanisms. Network pharmacology predicts interactions between active components and immune-related targets, while multi-omics validates these predictions at different molecular levels. This approach has been applied to complex TCM formulas and herbal medicines (191, 192). Metabolomics is particularly useful for studying immunometabolic reprogramming; for example, a TCM injection reduced MDSC and Treg levels while increasing CD8^+^ and CD4^+^ T cells by interfering with fatty acid metabolism in a melanoma brain metastasis model (193). Whether covalent modification of metabolic enzymes by electrophilic groups constitutes the metabolic basis of the immunoactivation window remains to be explored (33–35, 45–47).

Chemical biology approaches can confirm direct targets of active components. Drug affinity responsive target stability has identified cytosolic phospholipase A2 as a direct target of aconitine (194). Similar strategies can identify direct targets of cantharidin and triptolide, grounding toxicity-efficacy homology in specific protein-drug covalent binding events (45–47, 148–150).

### 8.2 Innovative drug design and combination strategy development

Intelligent responsive nano-delivery systems are a key direction for overcoming the bioavailability bottleneck of active components from toxic TCMs. Exploiting the differences between normal tissues and the TIME (weak acidity, high enzyme activity, high GSH concentration), pH-responsive nanoparticles can be designed to trigger release in the acidic tumor microenvironment, or redox-responsive systems can be constructed to utilize the high GSH concentration in tumor cells for disulfide bond cleavage and drug release (161, 165). Developing novel derivatives and PROTAC molecules based on structural biology and computer-aided design represents an innovative direction at the chemical level. YX-112, a celastrol-derived PROTAC designed to target degradation of CHEK1 and PIK3R2 proteins, showed three times higher antiproliferative activity against TNBC cells compared to the parent compound (195). Icaritin-based PROTACs identified Bax inhibitor-1 as a critical target in Burkitt lymphoma (148), and similar PROTAC strategies have been applied to other natural products (149, 150). AI technology provides powerful computational tools for the rational design of toxic TCMs, while deep learning can analyze structure-activity relationships to predict targets and toxicity, and multi-omics integration can optimize drug combinations and timing (196). Regarding optimization of combination therapy strategies, sequential therapy using low-dose triptolide or arsenic first to remodel the TIME, followed by ICIs, has shown superior synergy compared to concurrent administration in several studies (14, 90, 105, 113). Concurrent combination, such as synchronized administration of cantharidin nanomedicine with PD-1 blockade to enhance antigen presentation via ICD (16), is suitable for components where ICD is the core mechanism. Alternating therapy could be used to avoid resistance, suitable for scenarios requiring long-term management. Integrating the “sovereign, minister, assistant, guide” (Jun-Chen-Zuo-Shi) principle of TCM formulation with modern combination therapy concepts could lead to more systematic design principles.

### 8.3 High-quality clinical research and translation pathways

Conducting rigorous Phase I/II clinical trials to systematically evaluate safety, determine the maximum tolerated dose, and explore biomarkers for combination with immunotherapy is a necessary path for the clinical translation of toxic TCMs. The phase II study of icaritin in advanced HBV-related HCC, which used strict inclusion/exclusion criteria and incorporated immunodynamic biomarkers, provides a referenceable paradigm for the clinical development of other active components from toxic TCMs (125). Biomarker-guided precision medicine is key to individualized toxic TCM therapy. Combining the patient’s tumor immune phenotype with multidimensional biomarkers such as tumor mutational burden, ratios of specific immune cell subsets (e.g., CD8^+^ T-cell/Treg ratio), cytokine profiles (e.g., IL-6, IL-10), and gut microbiota composition to build predictive models will help screen populations most likely to benefit from specific toxic TCM therapies (1–5, 87, 91, 116, 125). Baseline IL-6 levels and dynamic changes in serum cytokines for icaritin have been preliminarily shown to have predictive value for efficacy (125). Real-time monitoring of immune markers in the blood (e.g., PD-1 expression, Treg proportion) could help dynamically adjust the dose and regimen of toxic TCMs (197). Conducting multicenter, randomized, double-blind, placebo-controlled high-quality clinical trials that integrate immunological endpoints with traditional efficacy endpoints and safety endpoints is the only way to obtain high-level clinical evidence. One of the largest knowledge gaps in this field currently is the lack of prospective study designs that systematically correlate TCM syndrome differentiation with modern immune phenotyping.

### 8.4 Mediating role of the gut microbiota-immunity axis

Oral administration of toxic TCMs inevitably leads to interactions with the gut microbiota, and may profoundly impact inter-individual differences in drug efficacy. Orally administered icariin is hydrolyzed by gut microbiota to icariside II and further to icaritin, so microbiota composition determines the active form and systemic exposure (116). Icariside I reshapes the gut microbial community structure in a melanoma mouse model, significantly restoring abundance of beneficial bacteria such as *Lactobacillus* and *Bifidobacterium*, increasing short-chain fatty acid levels, and upregulating multiple lymphocyte subsets in peripheral blood, including CD4^+^, CD8^+^ T cells, and NK, NKT cells (198). These findings indicate that the gut microbiota-immunity axis is an important mediator of the systemic immunomodulatory effects of toxic TCMs.

Future research should directly investigate the impact of toxic TCMs and their metabolites on the composition and function of the gut microbiota, identify key microbiota-derived metabolites (e.g., short-chain fatty acids, bile acids, tryptophan metabolites), and elucidate how these changes systemically affect tumor development, progression, and treatment response via immune, neural, or endocrine pathways. A particularly noteworthy hypothesis is that differences in microbiota composition among individuals may be an important variable determining the location and width of the immunoactivation window for toxic TCMs, offering a new explanatory dimension for understanding inter-individual differences in clinical efficacy (116, 198).

### 8.5 Precision regulation driven by new technologies

Organ-on-a-chip technology combines patient-derived organoids with microfluidics to simulate TIME dynamics and drug delivery, which can be used to evaluate the efficacy of active components from toxic TCMs against patient-specific tumors, potentially enabling personalized drug screening (111). “Window-of-opportunity” clinical trials, where short-term drug administration is followed by tissue analysis to rapidly assess the drug’s impact on the TIME, are effective strategies for identifying predictive biomarkers and are also applicable to toxic TCMs (199). Network pharmacology and graph neural networks can be used for target prediction, QSAR models can predict toxicity of structurally modified derivatives, and machine learning can recommend optimal combination regimens and timing (196). AI technology provides powerful computational tools for the rational design of toxic TCMs, and deep learning can analyze structure-activity relationships to predict targets and toxicity (200). AI models may also integrate *in vitro* ICD data, *in vivo* toxicity data, and pharmacokinetic parameters to predict the safe dose range of the immunoactivation window for each toxic TCM component, thereby accelerating the transition from empirical screening to rational design (109).

Epigenetic modifications have emerged as an important dimension of toxic TCM action. Arsenic induces DNA methylation changes that may affect PD-L1 expression (201), and cantharidin suppresses the EZH2/H3K27me3 axis, thereby controlling cell cycle progression and antitumor immunity (101). Consistently, oxymatrine downregulates PD-L1 by promoting DNA demethylation (28). At low doses, the N→O bond of oxymatrine functions as a mild epigenetic modulator, providing a mechanistic explanation for toxicity-efficacy homology. An open question is whether epigenetic regulation mediates the “memory” of hormesis, by which subtoxic doses epigenetically reprogram immune cells to mount sustained antitumor responses.

Beyond epigenetic regulation, synthetic biology offers a promising approach for the efficient and green production of active components from toxic TCMs. The complete biosynthesis of icaritin using engineered *Saccharomyces cerevisiae* and *Escherichia coli* has already been achieved (202), paving the way for large-scale, sustainable manufacturing. Collectively, the convergence of these emerging fields, including multi-omics and AI-assisted design, gut microbiota-immunity axis, organ-on-a-chip technology, and synthetic biology, will accelerate the modernization and evidence-based translation of toxic TCMs in cancer immunotherapy.

## 9 Conclusion

Toxic TCMs possess a distinctive, systems-level capacity to remodel the TIME through multiple targets and pathways. Their core mechanisms span the entire cancer-immunity cycle, from inducing immunogenic cell death and reprogramming tumor-associated macrophages to promoting CD8^+^ T-cell infiltration and downregulating PD-L1. This comprehensive immune-remodeling capability offers new strategies for overcoming resistance to existing immunotherapies and improving response rates. The concept of toxicity-efficacy homology distinguishes toxic TCMs from conventional cytotoxic agents by emphasizing that reactive chemical moieties can serve simultaneously as the structural basis for immunological activity and the source of organ toxicity. Through dose-dependent bidirectional regulation, low doses may trigger adaptive stress responses that activate antitumor immunity, whereas high doses cause non-specific toxicity. Defining the immunoactivation window for each component therefore represents a pivotal scientific challenge for clinical translation. This framework is further supported by epigenetic modulation and by chemical-level toxicity-efficacy separation within the same herb (28, 30). Even components with low intrinsic toxicity, such as *Epimedium* flavonoids and centipede peptides, which lack strong electrophilic groups, maintain an efficacy/toxicity balance through dose-dependent signaling modulation, illustrating the broader applicability of the toxicity-efficacy homology framework (31–35).

The therapeutic value of toxic TCMs extends beyond direct cytotoxicity to systemic, multi-target regulation of the TIME, complementing the single-target focus of current immunotherapies. Translating this potential requires overcoming a narrow therapeutic window, chemical complexity, formulation limitations, and insufficient quality control. Structural modification and prodrug design, stimuli-responsive nano-delivery, multi-omics and systems pharmacology, biomarker-stratified clinical trials, and investigation of the gut microbiota-immunity axis represent key priorities (116, 125, 148–150, 161, 165, 190, 191, 198). Future progress will depend on interdisciplinary convergence, including AI-assisted design (196, 200), single-cell and spatial omics (106–109), organ-on-a-chip and humanized models (111), and synthetic biology (202). A precision combination framework grounded in tumor immune phenotyping can inform clinical trial design and help translate “combating poison with poison” into an evidence-based paradigm of “attenuated toxicity and enhanced efficacy”.

## Glossary

13-OD: 13-oxyingenol dodecanoate
ACCS: ATO combined with cryptotanshinone
AE: adverse event
AhR: aryl hydrocarbon receptor
AI: artificial intelligence
AKT: protein kinase B
AMPK: AMP-activated protein kinase
APL: acute promyelocytic leukemia
ATO: arsenic trioxide
ATRA: all-trans retinoic acid
ATP: adenosine triphosphate
Bcl-2: B-cell lymphoma 2
BCRP: breast cancer resistance protein
BTB: broad-complex, tramtrack, and bric-à-brac
CAF: cancer-associated fibroblast
CAR-T: chimeric antigen receptor T cell
CCL2: C-C motif chemokine ligand 2
CCR2: C-C motif chemokine receptor 2
CD: cluster of differentiation
cGAS: cyclic GMP-AMP synthase
CKI: Compound Kushen Injection
CRT: calreticulin
CSF1R: colony-stimulating factor 1 receptor
CTL: cytotoxic T lymphocyte
CTLA-4: cytotoxic T-lymphocyte-associated protein 4
CXCL: C-X-C motif chemokine ligand
CXCR4: C-X-C chemokine receptor type 4
CYP: cytochrome P450
DAMP: damage-associated molecular pattern
DC: dendritic cell
DCR: disease control rate
DFS: disease-free survival
DLAT: dihydrolipoamide S-acetyltransferase
DR4/DR5: death receptor 4/5
ECM: extracellular matrix
EGFR: epidermal growth factor receptor
EMT: epithelial-mesenchymal transition
EPR: enhanced permeability and retention
ER: endoplasmic reticulum
ERK: extracellular signal-regulated kinase
ESCC: esophageal squamous cell carcinoma
EZH2: enhancer of zeste homolog 2
FADD: Fas-associated protein with death domain
FAK: focal adhesion kinase
FNPS: Fuzi neutral polysaccharide
GSDMD: gasdermin D
GSDME: gasdermin E
GSH: glutathione
GSK-3β: glycogen synthase kinase-3 beta
GPX4: glutathione peroxidase 4
HCC: hepatocellular carcinoma
HIF-1α: hypoxia-inducible factor 1-alpha
HILIC: hydrophilic interaction liquid chromatography
HMGB1: high mobility group box 1
HSP90β: heat shock protein 90 beta
HUVEC: human umbilical vein endothelial cell
ICD: immunogenic cell death
ICI: immune checkpoint inhibitor
IDO1: indoleamine 2, 3-dioxygenase 1
IFN-γ: interferon-gamma
IKK-α: inhibitor of nuclear factor kappa-B kinase subunit alpha
IL: interleukin
iRECIST: immune Response Evaluation Criteria in Solid Tumors
irAE: immune-related adverse event
ISC: ion suppression chromatography
JAK: Janus kinase
KEAP1: kelch-like ECH-associated protein 1
KPS: Karnofsky performance status
LAG-3: lymphocyte activation gene-3
LBP-4: Lycium barbarum polysaccharide-4
LLDT-8: (5R)-5-hydroxytriptolide
LOXL3: lysyl oxidase-like 3
MACC1: metastasis-associated in colon cancer 1
MAPK: mitogen-activated protein kinase
MDSC: myeloid-derived suppressor cell
MDR: multidrug resistance
MHC-I: major histocompatibility complex class I
MME: membrane metalloendopeptidase
MRD: minimal residual disease
MRP1: multidrug resistance-associated protein 1
MTD: maximum tolerated dose
mTOR: mammalian target of rapamycin
NF-κB: nuclear factor kappa-light-chain-enhancer of activated B cells
NK: natural killer
NKT: natural killer T
NOAEL: no observed adverse effect level
NSCLC: non-small cell lung cancer
ORR: objective response rate
OS: overall survival
OSCC: oral squamous cell carcinoma
PARP: poly(ADP-ribose) polymerase
PBMC: peripheral blood mononuclear cell
PCBP1/2: poly(rC)-binding protein 1/2
PD-1: programmed death-1
PD-L1: programmed death-ligand 1
PFS: progression-free survival
P-gp: P-glycoprotein
PI3K: phosphoinositide 3-kinase
PML-RARα: promyelocytic leukemia-retinoic acid receptor alpha
PP2A: protein phosphatase 2A
PROTAC: proteolysis targeting chimera
PSC: pancreatic stellate cell
QC: quality control
QSAR: quantitative structure-activity relationship
RCD: regulated cell death
RECIST: Response Evaluation Criteria in Solid Tumors
RGD: arginylglycylaspartic acid
ROS: reactive oxygen species
SCFA: short-chain fatty acid
scRNA-seq: single-cell RNA sequencing
SHP2: Src homology 2 domain-containing phosphatase 2
SIRT3: sirtuin 3
SPF: specific pathogen-free
STAT: signal transducer and activator of transcription
STING: stimulator of interferon genes
TAM: tumor-associated macrophage
TCM: traditional Chinese medicine
TGF-β: transforming growth factor-beta
TIL: tumor-infiltrating lymphocyte
TIM-3: T-cell immunoglobulin and mucin domain-containing protein 3
TIME: tumor immune microenvironment
TLR4: Toll-like receptor 4
TMB: tumor mutational burden
TNF-α: tumor necrosis factor-alpha
Treg: regulatory T cell
TTP: time to progression
UHPLC-Q-TOF/MS: ultra-high-performance liquid chromatography-quadrupole time-of-flight mass spectrometry
VEGF: vascular endothelial growth factor
WGS: whole-genome sequencing
XRD: X-ray diffraction
